# Molecular genotyping reveals multiple carbapenemase genes and unique *bla*_OXA-51-like_ (*oxaAb*) alleles among clinically isolated *Acinetobacter baumannii* from a Philippine tertiary hospital

**DOI:** 10.1186/s41182-024-00629-w

**Published:** 2024-09-26

**Authors:** Mark B. Carascal, Raul V. Destura, Windell L. Rivera

**Affiliations:** 1 Pathogen-Host-Environment Interactions Research Laboratory, Institute of Biology, College of Science, University of the Philippines Diliman, 1101 Quezon City, Philippines; 2 Clinical and Translational Research Institute, The Medical City, Ortigas Avenue, 1605 Pasig City, Philippines; 3 Institute of Molecular Biology and Biotechnology, National Institutes of Health, University of the Philippines, 1159 Manila, Philippines

**Keywords:** β-lactamase, *Acinetobacter baumannii*, Carbapenemase, Carbapenem resistance, *oxaAb*, Philippines

## Abstract

**Background:**

*Acinetobacter baumannii* continued to be an important Gram-negative pathogen of concern in the clinical context. The resistance of this pathogen to carbapenems due to the production of carbapenemases is considered a global threat. Despite the efforts to track carbapenemase synthesis among *A. baumannii* in the Philippines, local data on its molecular features are very scarce. This study aims to characterize *A. baumannii* clinical isolates from a Philippine tertiary hospital through genotyping of the pathogen’s carbapenemase genes.

**Methods:**

Antibiotic susceptibility profiling, phenotypic testing of carbapenemase production, and polymerase chain reaction assays to detect the different classes of carbapenemase genes (class A *bla*_KPC_, class B *bla*_NDM_, *bla*_IMP_, *bla*_VIM_, and class D *bla*_OXA-23-like_, *bla*_OXA-24/40-like_, *bla*_OXA-48-like_, *bla*_OXA-51-like_, *ISAba1-bla*_OXA-51-like_, *bla*_OXA-58-like_) were performed in all collected *A. baumannii*, both carbapenem resistant and susceptible (*n* = 52).

**Results:**

Results showed that the majority of the carbapenem-resistant strains phenotypically produced carbapenemases (up to 84% in carbapenem inactivation methods) and possessed the *ISAba1-bla*_OXA-51-like_ gene complex (80%). Meanwhile, both carbapenem-resistant and carbapenem-susceptible isolates possessed multi-class carbapenemase genes including *bla*_NDM_ (1.9%), *bla*_VIM_ (3.9%), *bla*_OXA-24/40-like_ (5.8%), *bla*_OXA-58-like_ (5.8%), *bla*_KPC_ (11.5%), and *bla*_OXA-23-like_ (94.2%), which coexist with each other in some strains (17.3%). In terms of the intrinsic *bla*_OXA-51-like_ (*oxaAb*) genes, 23 unique alleles were reported (*bla*_OXA-1058_ to *bla*_OXA-1080_), the majority of which are closely related to *bla*_OXA-66_. Isolates possessing these alleles showed varying carbapenem resistance profiles.

**Conclusions:**

In summary, this study highlighted the importance of molecular genotyping in the characterization of *A. baumannii* by revealing the carbapenemase profiles of the pathogen (which may not be captured accurately in phenotypic tests), in identifying potent carriers of transferrable carbapenemase genes (which may not be expressed straightforwardly in antimicrobial susceptibility testing), and in monitoring unique pathogen epidemiology in the local clinical setting.

**Supplementary Information:**

The online version contains supplementary material available at 10.1186/s41182-024-00629-w.

## Background

*Acinetobacter baumannii* is a notorious pathogen implicated in serious nosocomial infections worldwide. Among the choice antibiotics for treating severe *A. baumannii* infections are carbapenems (imipenem and meropenem) [1]. However, global surveillance studies found an increase in the occurrence of carbapenem-resistant *A. baumannii* (CRAb), particularly among low- to middle-income countries [2]. In Southeast Asia (SEA), a region considered a focal point of global antimicrobial resistance, CRAb emerges as an imminent threat [3]. For instance, the Philippine health authority recently stated that more than 50% of the local *A. baumannii* isolates are already resistant to imipenem and meropenem [4]. Infections due to CRAb have very limited treatment options and were associated with increased hospitalization, in-patient costs, and mortality [5]. As a result, the World Health Organization (WHO) identified CRAb as a critical priority pathogen requiring new antimicrobials [6].

The mechanisms of carbapenem resistance in *A. baumannii* include carbapenemase production, efflux pumps, and porin loss. Among these pathways, the synthesis of carbapenemases is the most prevalent [7]. Carbapenemases are types of β-lactamases under molecular classes A (serine β-lactamases), B (metallo-β-lactamases), and D (oxacillinases) with carbapenem-hydrolyzing capabilities. Different acquired carbapenemase genes have been reported for *A. baumannii* in the last decade such as Ambler class A *bla*_KPC_ [8], class B *bla*_NDM_ [9], *bla*_VIM_ [10], *bla*_IMP_ [11], class D *bla*_OXA-23-like_ [12], *bla*_OXA-24/40-like_ [13], *bla*_OXA-58-like_ [14], and *bla*_OXA-143-like_ [15]. The class D *bla*_OXA-51-like_ (recently referred to as *oxaAb*) is considered intrinsic β-lactamases in *A. baumannii*, with significant carbapenemase activity observed in the presence of upstream insertion sequences such as *ISAba1* [16].

Clinical isolates of CRAb are profiled for carbapenemase genes given the active spread of transferrable β-lactamases among Gram-negative pathogens [17]. Molecular surveillance of CRAb carbapenemases in SEA has been reported previously [18, 19]. Meanwhile, the Philippine Antimicrobial Resistance Surveillance Program recently released the first comprehensive genomic analysis of 104 CRAb isolates in the country [20]. Despite these efforts, the detection of carbapenemases in the clinical setting remains highly reliant on phenotypic tests. Unfortunately, current tests are not reliable when utilized in *A. baumannii* [21, 22]. As the clinical management and epidemiology of CRAb are dependent on its mechanism of resistance, molecular genotyping remains to be the gold standard in inferring carbapenemase production through the detection of carbapenemase genes. In this investigation, we revealed the presence of multiple carbapenemase genes in clinically isolated *A. baumannii* [CRAb and carbapenem-susceptible *A. baumannii* (CSAb)] and linked them to the pathogen’s antibiotic resistance profiles and carbapenemase production phenotypes. In addition, we described 23 novel *bla*_OXA-51-like_ gene alleles, indicating the unique molecular epidemiology of Philippine CRAb isolates necessitating further surveillance.

## Methods

### Collection and characterization of the isolates

Stock cultures of non-duplicate *A. baumannii* clinical isolates from October 2018 to September 2020 (isolated from different patients with clinically significant infections) were collected from the Section of Clinical Microbiology of The Medical City, Pasig City, Philippines. No identifiable patient-related information was gathered; only microbiological and sample-related data were collected. A total of 52 clinically significant isolates were successfully collected. The original isolation sources include endotracheal aspirate (*n* = 15), sputum (*n* = 13), blood (*n* = 11), urine (*n* = 6), wound (*n* = 6), and abscess (*n* = 1). All isolates were maintained in blood agar medium (Thermo Scientific™, USA) incubated at 35 °C–37 °C. Isolates were identified using Vitek^®^ MS Matrix-Assisted Laser Desorption/Ionization-Time of Flight (MALDI-TOF; bioMérieux, France), while the antibiotic susceptibility profiles were generated using Vitek^®^ 2 Compact Analyzer (bioMérieux, France) with Vitek^®^ 2 Advanced Expert System (AES, bioMérieux, France). The antibiotics examined include ceftazidime, ciprofloxacin, ceftriaxone, cefepime, gentamicin, imipenem, meropenem, trimethoprim-sulfamethoxazole, and piperacillin–tazobactam. Carbapenem resistance was determined using the latest recommended minimum inhibitory concentration (MIC) cutoff values from the Clinical and Laboratory Standards Institute (CLSI) [22]. Quality check was done through the routine assay performance testing as conducted by the clinical laboratory (accredited by the local health department) and using CLSI-recommended standard strains for Gram-negative bacteria (*E. coli* ATCC^®^ 25,922™ and *P. aeruginosa* ATCC^®^ 27,853™) [22]. The MICs of the isolates to carbapenems are described in Additional File 1.

### Detection of carbapenemase production

All isolates underwent phenotypic assessment of carbapenemase production using the modified Hodge test (MHT), modified carbapenem inactivation method (mCIM), and Tris-modified carbapenem inactivation method (CIM-Tris). For MHT, the protocol of Amjad et al. was used [23]. Briefly, 18–24-h-old isolates were heavily streaked from the edge of 10 µg ertapenem (ERT) disks (Mastdiscs^®^ AST, UK) placed on Mueller–Hinton agar (MHA, Himedia^®^, India) with a lawn of 0.5 McFarland-standardized *Escherichia coli* ATCC^®^ 25,922™. The cloverleaf-like zone of inhibition (ZOI) within the streak indicated carbapenemase production. Meanwhile, mCIM was carried out using the procedure of Pierce et al. [24]. Briefly, 1µL loopful of 18–24-h-old isolates was suspended in 2 mL Tryptic Soy Broth (TSB, Himedia^®^, India) with 10 µg meropenem (MEM) disk (Mastdiscs^®^ AST, UK) and incubated at 35 °C for 4 h. After incubation, the disks were harvested and put on MHA plates with 0.5 McFarland-standardized *E. coli* ATCC^®^ 25,922™. Isolates with ZOI of 6–10 mm were reported as carbapenemase producers. Finally, CIM-Tris was completed utilizing the methodology of Uechi et al. [25]. In summary, 10µL loopful of overnight isolate cultures was suspended in 400µL of 0.5 M Tris–HCl buffer at pH 7.6 (Trizma^®^, Germany) with 10 µg MEM disk (Mastdiscs^®^ AST, UK) and incubated at 35 °C for 2 h. The ensuing steps were the same as specified for mCIM. Isolates with ZOI of 6–15 mm were reported as carbapenem producers. All setups were carried out in triplicates and incubated at 35 °C–37 °C for 18–24 h prior to ZOI measurements. For all experiments (including the replicates), the quality control strains recommended by the CLSI were used: *Klebsiella pneumoniae* ATCC^®^ BAA-1705™ (carbapenemase positive) and ATCC^®^ BAA-1706™ (carbapenemase negative) [22]. Meropenem disk batches used in the assays were also quality-checked through routine disk diffusion in *E. coli* ATCC^®^ 25,922™, as recommended, prior to usage.

### Detection of carbapenemase genes

Genomic DNA from each isolate was extracted using the boil lysis procedure [26] and measured using Nanodrop™ 2000 Spectrophotometer (Thermo Fisher Scientific™, USA). Primers targeting class A (*bla*_KPC_), class B (*bla*_NDM_, *bla*_IMP_, *bla*_VIM_), and class D (*bla*_OXA-23-like_, *bla*_OXA-24/40-like_, *bla*_OXA-48-like_, *bla*_OXA-51-like_, *ISAba1-bla*_OXA-51-like_, *bla*_OXA-58-like_) carbapenemase genes were used for the polymerase chain reaction (PCR; Additional File 1). PCR tests were carried out in 25 µL single reactions (with 12.5 µL GoTaq^®^ Green Master Mix (Promega, USA), 1.0 µL of 10 µM primers, 50–100 ng DNA sample, and nuclease-free water) using a GeneExplorer™ thermal cycler (Bioer, China) following the PCR procedures listed in Additional File 1. Gel electrophoresis of the amplicons was performed in 1.8%–2% agarose (1st BASE, Singapore) on a MyGel Instaview^®^ gel electrophoresis system (Accuris, Korea) set at 135 V for 30 min, with 1X Tris–acetate-EDTA running buffer (Vivantis, Malaysia). The final gels were visualized and documented in E-Gel™ Power Snap electrophoresis system (Invitrogen™, USA). All PCR assays were done in two trials using appropriate controls. The expected band size of the amplicons and the controls used for genotyping were outlined in Additional File 1.

### Sequence-based clonal typing using *bla*_OXA-51-like_ genes

Sequence-based typing (SBT) of *bla*_OXA-51-like_ genes was performed to determine the major clonal lineages of the isolates, conforming with the traditional multilocus sequence typing [27–29]. In brief, all *bla*_OXA-51-like_ amplicons from the isolates, together with an amplicon from *A. baumannii* ATCC^®^ BAA-1605 as a control, underwent paired-end Sanger sequencing (Macrogen, Korea). Complete consensus DNA from the forward and reverse sequences and their matching amino acid sequences were generated using Bioedit version 7.2.5. Initial identities of the DNA and amino acid sequences were determined using the Basic Local Alignment Search Tool incorporated in the Beta-Lactamase Database-Structure and Function (BLDB; http://www.bldb.eu/; accessed on 10 April 2023). Sequences with 100% matching protein identities with known *bla*_OXA-51-like_ variants (with or without silent single nucleotide mutations) were reported as the native matching allele. Meanwhile, sequences with at least one mismatch in amino acid sequence compared with all known *bla*_OXA-51-like_ variants were described as novel alleles following the recent recommendations in β-lactamase reporting and nomenclature [30]. An unrooted maximum likelihood tree of the *bla*_OXA-51-like_ genes was created in PhyML version 3.1 following the parameters derived from jModelTest version 20,160,303 and using 87 reference *bla*_OXA-51-like_ gene sequences (Additional File 1) from various subclades of the OXA-51 subfamily tree (http://www.bldb.eu/alignment.php?align=D:OXA-51-like; accessed on 10 April 2023). Changes in amino acids were manually mapped from the multiple sequence alignment of the alleles using Clustal W in Bioedit version 7.2.5, with *bla*_OXA-66_ as the leader sequence. All original annotated sequences were submitted to GenBank (https://www.ncbi.nlm.nih.gov/genbank/; accessed on 15 March 2023) with accession numbers OM617740-OM617792 (Additional File 1). The unique *bla*_OXA-51-like_ alleles are currently annotated as antimicrobial resistance reference genes in GenBank under Bioproject number PRJNA313047 (reference sequence numbers NG_079900-NG_079922) and are also accessible at the BLDB (http://www.bldb.eu/; accessed on 10 April 2023).

### Data analysis and visualization

Phenotypic data including antibiotic susceptibility profiles and carbapenemase production were visualized in a heatmap using Microsoft Office 365 Excel (Microsoft, USA). Genotypic data such as gel electrophoretograms and genotype listing were compiled in Microsoft Office 365 PowerPoint (Microsoft, USA). Amino acid linkage map was made in Microsoft Office 365 PowerPoint (Microsoft, USA) following the model of Evans et al. [31]. Statistical analysis on the sensitivity and specificity of phenotypic tests in detecting carbapenemase production compared to the carbapenem resistance profile of the isolates was computed using the MedCalc version 20.218 (https://www.medcalc.org/calc/diagnostic_test.php; accessed 15 January 2023). Results were reported as 95% confidence intervals whenever appropriate.

## Results

### A significant number of isolates are carbapenemase-producing CRAb

Of the 52 collected, clinically significant *A. baumannii* isolates, 25 (48.1%) were considered CRAb based on the Vitek^®^ 2 Compact and Advanced Expert System (AES) results, with their corresponding minimum inhibitory concentrations (MICs) described in Additional File 1. All CRAb has an MIC of ≥ 16 mg/L against meropenem and imipenem, whereas CSAb isolates have MICs ranging from ≤ 0.25 to 1 mg/L. All CRAb isolates were considered multidrug resistant (i.e., resistant to at least two antibiotic classes), with 22 (88.0%) being non-susceptible (resistant and intermediately resistant) to all antibiotics tested. Meanwhile, from the CSAb, three (11.1%) were considered multidrug resistant, with resistance ranging from cephalosporins, aminoglycosides, folate pathway antagonists, and β-lactam combination agents. Only six (11.5%) of the total isolates were susceptible to all antibiotics tested.

Results from several phenotypic tests for carbapenemase production presented variabilities. Among the CRAb, carbapenemase production was detected in 13 (52%), 18 (72%), and 21 (84%) isolates using the modified Hodge test (MHT), modified carbapenem inactivation method (mCIM), and Tris-modified carbapenem inactivation method (CIM-Tris), respectively. Meanwhile, carbapenemase production was found in 9 (33.3%) and 12 (44.4%) carbapenem-susceptible isolates using mCIM and CIM-Tris, respectively. Carbapenemase production was not reported in any carbapenem-susceptible isolates using MHT. The concordance of the phenotypic assays with the carbapenem resistance profiles showed the highest sensitivity in CIM-Tris [84.0% (63.9%–95.5%)] and the highest specificity in MHT [100% (87.2%–100%)] (Table 1).

**Table 1:** Comparison among the results of the modified Hodge test (MHT), modified carbapenem inactivation method (mCIM), and Tris-modified carbapenem inactivation method (CIM-Tris) relative to the carbapenem-resistant (CRAb) and carbapenem-susceptible (CSAb) antibiotic susceptibility testing (AST) of the clinical *A. baumannii* isolates in Vitek^®^ 2 Compact with Advanced Expert System (bioMérieux, France)

Isolates (Carbapenemase production phenotype)	MHT *n* (%)	mCIM *n* (%)	CIM-Tris *n* (%)
CRAb, *n* = 25 (carbapenemase positive)	13 (52)	18 (72)	21 (84)
CRAb, *n* = 25 (carbapenemase negative)	12 (48)	6 (24)*	4 (16)
CSAb, *n* = 27 (carbapenemase positive)	0	9 (33.3)	12 (44.4)
CSAb, *n* = 27 (carbapenemase negative)	27 (100)	13 (48.1)*	15 (55.6)
Analytical sensitivity (vs. AST results) [95% confidence interval]	52.0% (31.3%–72.2%)	75.0% (53.3%–90.2%)	84.0% (63.9%–95.5%)
Analytical specificity (vs. AST results) [95% confidence interval]	100% (87.2%–100%)	59.1% (36.4%–79.3%)	55.6% (35.3%–74.5%)

*The remaining isolates (to complete the total) were interpreted to have “indeterminate” results based on the interpretation criteria of Pierce et al. [24]

### *A. baumannii* possessed multiple carbapenemase genes

**Fig. 1:**
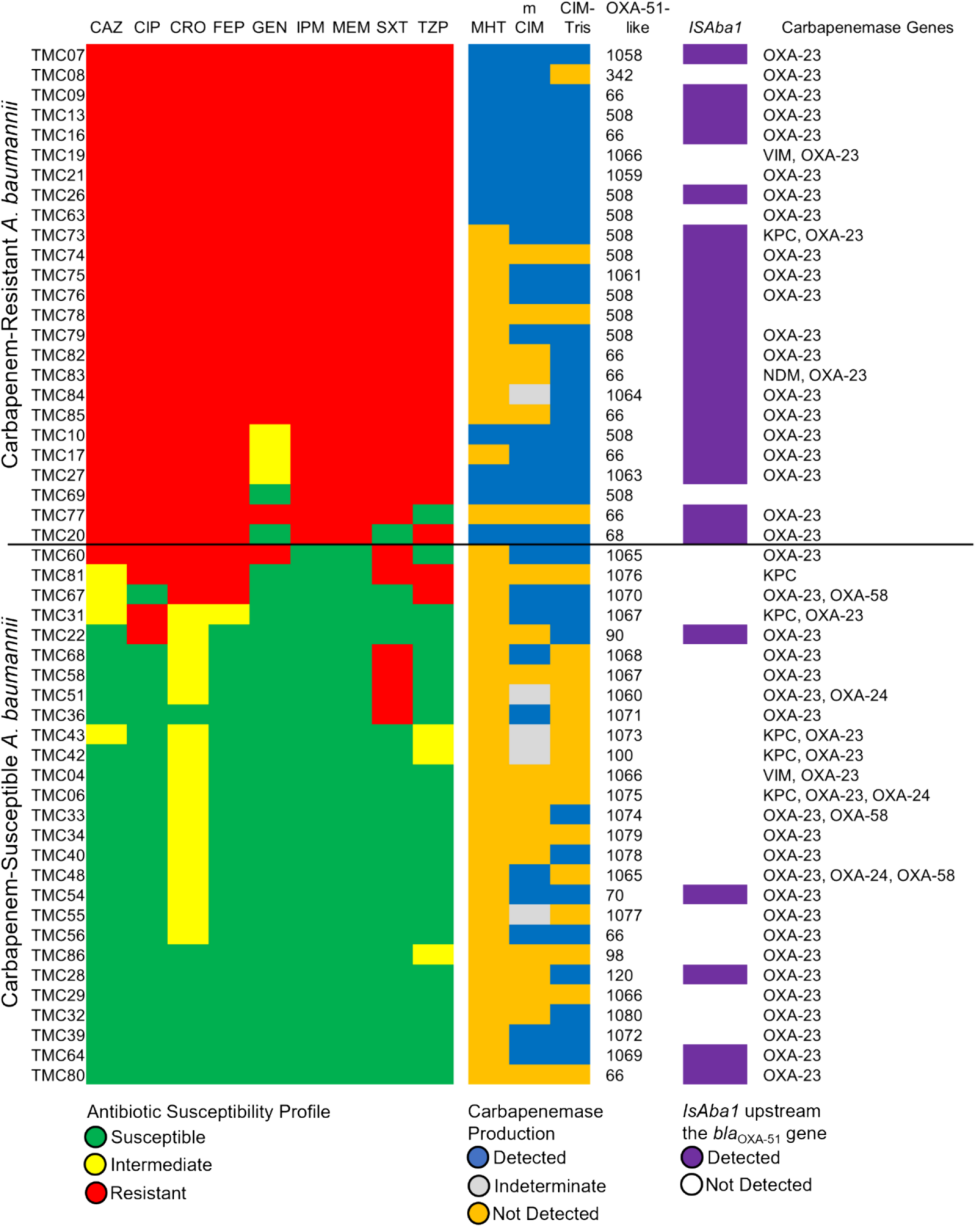
Summary of *A. baumannii* isolate characteristics based on their antibiotic susceptibility profiles in Vitek^®^ 2 Compact with Advanced Expert System (antibiotics used: CAZ, ceftazidime; CIP, ciprofloxacin; CRO, ceftriaxone; FEP, cefepime; GEN, gentamicin; IPM, imipenem; MEM, meropenem; SXT, trimethoprim-sulfamethoxazole; TZP, piperacillin–tazobactam), carbapenemase production (MHT, modified Hodge test; mCIM, modified carbapenem inactivation method; CIM-Tris, Tris-modified carbapenem inactivation method), type of *bla*_OXA-51-like_ alleles detected, presence of *ISAba1* upstream the *bla*_OXA-51-like_ genes, and other carbapenemase genes detected through PCR (KPC- *Klebsiella pneumoniae* carbapenemase, NDM- New Delhi Metallo-β-lactamase, VIM- Verona Integron-associated Metallo-β-lactamase, OXA- Oxacillinase)

All isolates possessed at least one of the examined carbapenemase genes, with Ambler class D genes as the most prevalent (Fig. 1; Additional File 1). All isolates harbored the intrinsic *bla*_OXA-51-like_ genes, with 25 (48.1%) also possessing the upstream *ISAba1*. The majority [20/25 (80.0%)] of the isolates with *ISAba1-bla*_OXA-51-like_ genes were classified as CRAb.

Among the Ambler class A carbapenemase genes, *bla*_KPC_ was reported in six (11.5%) isolates. Meanwhile, *bla*_NDM_ [1 (1.92%)] and *bla*_VIM_ [2 (3.85%)] were the class B carbapenemase genes found. Among the class D carbapenemase genes (except *bla*_OXA-51-like_), *bla*_OXA-23-like_ dominated [49 (94.2%)]. Only three each of *bla*_OXA-24/40-like_ and *bla*_OXA-58-like_ (5.8%) genes were found, all of which were from CSAb. The *bla*_IMP_ and *bla*_OXA-48-like_ genes were not detected in our isolates even after repeat PCR assays using appropriate controls.

Multi-class carbapenemase genes (i.e., class D genes in combination with either *bla*_KPC_, *bla*_NDM_, or *bla*_VIM_) were detected in nine (17.3%) isolates, whereas *bla*_OXA-23-like_ in combination with *bla*_OXA-24/40-like_ or *bla*_OXA-58-like_ were detected in five (9.62%) isolates. Only three of the 25 CRAb isolates (12.0%) possessed multi-class carbapenemase genes.

### *A. baumannii* clinical isolates harbored unique *bla*_OXA-51-like_ alleles

**Fig. 2:**
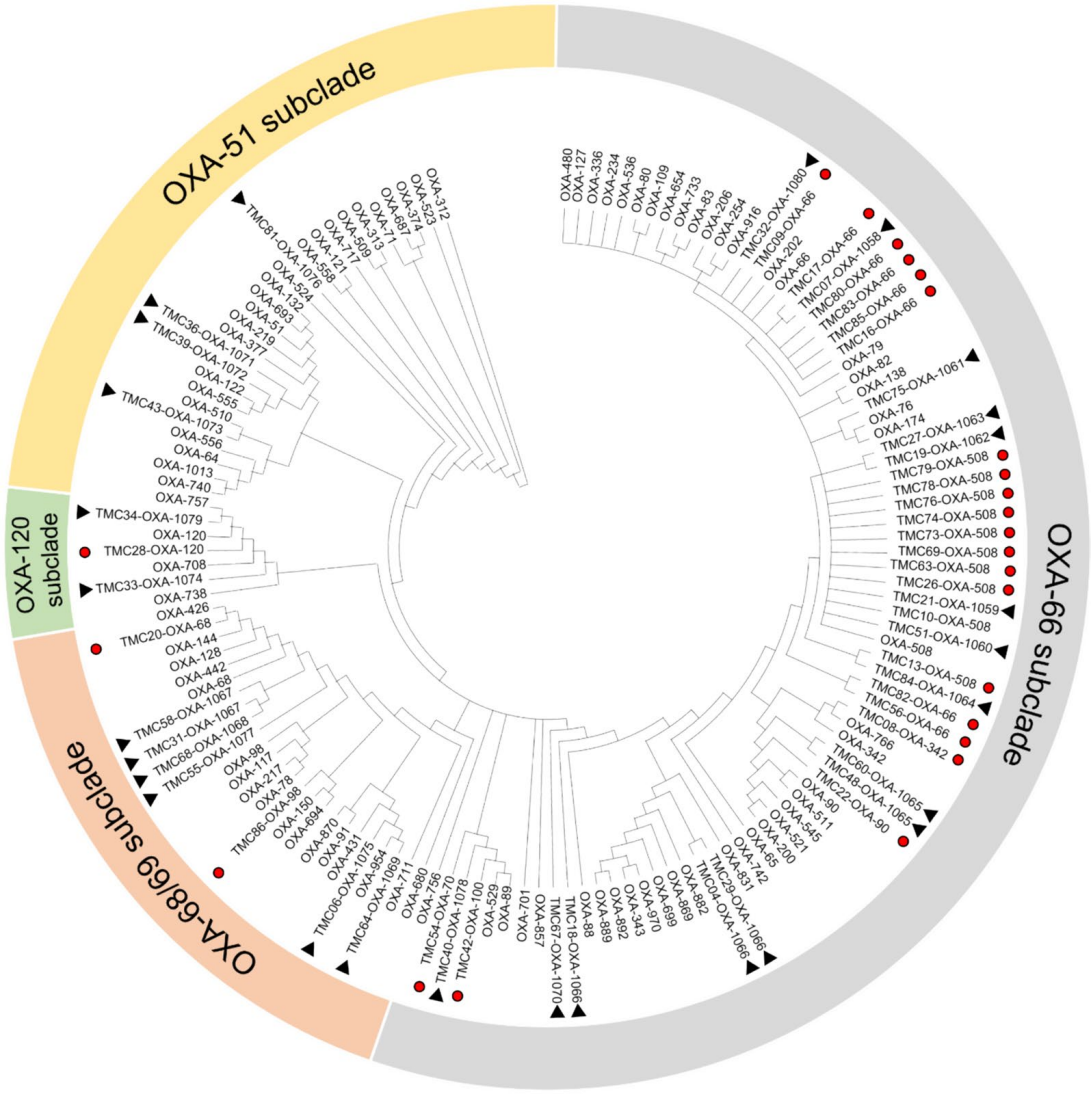
Unrooted maximum likelihood tree based on the 825 nucleotides of the *bla*_OXA-51-like_ genes of the study isolates and known references (Additional File 1) using the three-parameter (TPM1—unequal frequencies) with gamma distribution model of DNA substitution. Only the general tree topology was shown. Genes were clustered based on the subclades shown in the *bla*_OXA-51-like_ gene tree generated in the Beta-Lactamase Database-Structure and Function (http://www.bldb.eu/; accessed on 13 March 2023). Samples marked with red circles are alleles matching with known *bla*_OXA-51-like_ variants, while those with black triangles are the unique alleles. Most of the rare alleles clustered under the *bla*_OXA-66_ subclade under the international clonal complex 2 in sequence-based typing

Sequencing and phylogenetic analysis of the *bla*_OXA-51-like_ genes from the clinical isolates indicated that 25 (48.1%) had known *bla*_OXA-51-like_ alleles, with *bla*_OXA-508_ [10 (19.2%)] and *bla*_OXA-66_ [8 (15.4%)] as the most common (Fig. 2). One each of other known alleles such as *bla*_OXA-68_, *bla*_OXA-70_, *bla*_OXA-90_, *bla*_OXA-98_, *bla*_OXA-100_, *bla*_OXA-120_, and *bla*_OXA-342_ was also detected. The reference isolate, *A. baumannii* ATCC BAA-1605™, had the confirmed *bla*_OXA-69_ (GenBank accession number of OM617776). The rest of the isolates (*n* = 28; 53.8%) possessed unique *bla*_OXA-51-like_ alleles based on their amino acid sequences (Additional File 1). The majority [37 (71.2%)] of the discovered *bla*_OXA-51-like_ alleles clustered with the *bla*_OXA-66_ subclade [international clonal complex 2 in sequence-based typing (SBT)], while only three (5.8%), four (7.7%), and eight (15.4%) alleles clustered with *bla*_OXA-120_, *bla*_OXA-51_, and *bla*_OXA-68/69_ subclades (international clonal complex 1 in SBT), respectively (Fig. 2).

**Fig. 3:**
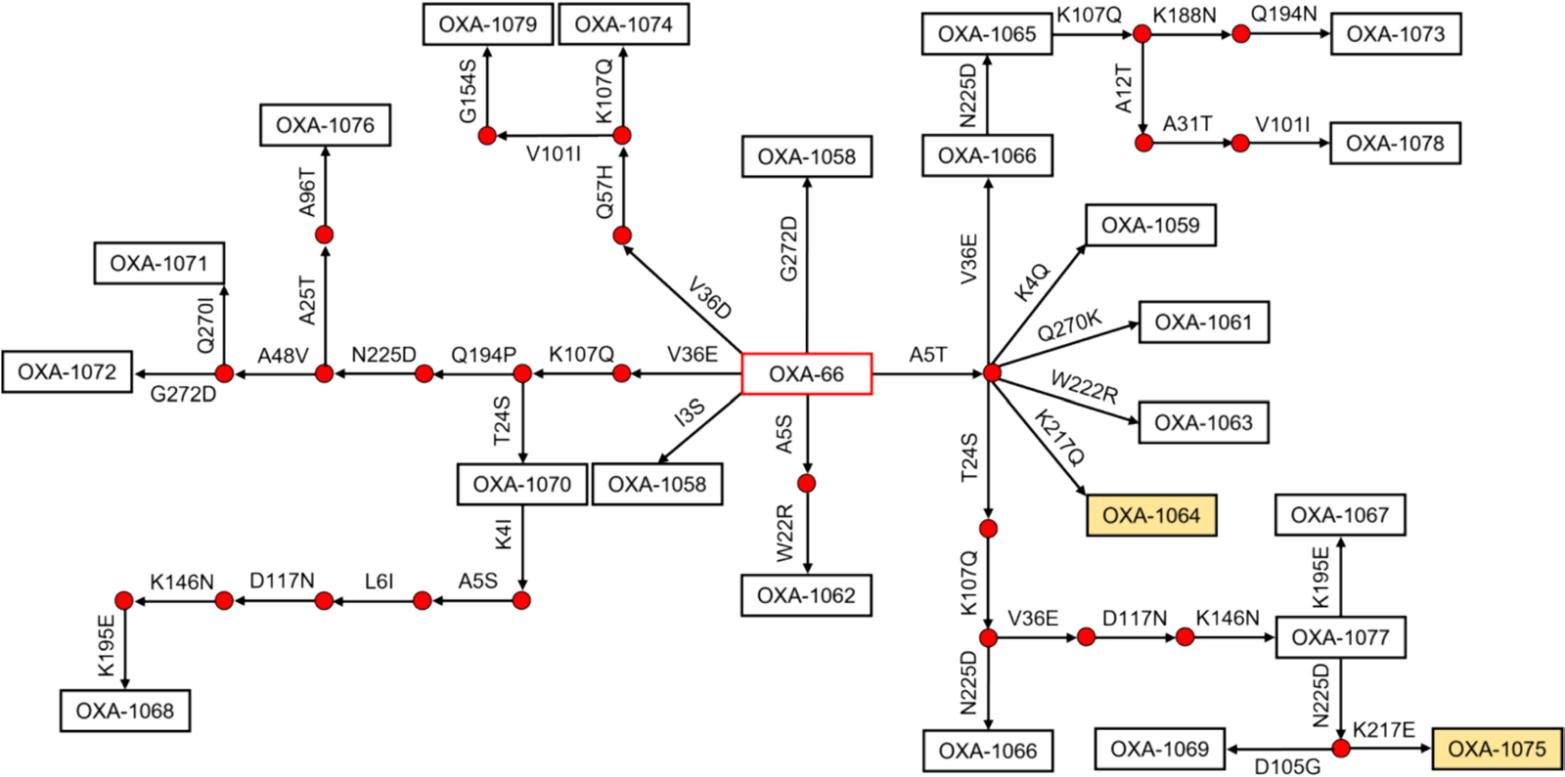
Amino acid linkage map of the unique *bla*_OXA-51-like_ alleles from the study. The amino acid substitution nomenclature indicates the original amino acid followed by the position and the replacement. The substitutions are relative to the reference *bla*_OXA-66_ sequence. Boxes colored yellow indicate alleles with amino acid changes in a predicted active site residue (K217)

The 23 new alleles reported were assigned as *bla*_OXA-1058_ to *bla*_OXA-1080_, with *bla*_OXA-1067_ and *bla*_OXA-1066_ detected in two (3.8%) and three (5.8%) isolates, respectively. Amino acid differences from the leader protein (*bla*_OXA-66_) range from one to nine (Fig. 3). The predicted conserved active site residues (S80, K83, S127, W166, K217, R260) [32] were unchanged for the new alleles, except for *bla*_OXA-1064_, and *bla*_OXA-1075_ with K217Q and K217E mutations, respectively (Additional File 1). Among the novel alleles, only five (21.7%) were found to have the upstream *ISAba1* (Fig. 1).

## Discussion

In the Philippines, the occurrence of CRAb has been independently reported [4]. Additionally, the genomic features of local CRAb isolates were recently characterized [20]. Our study contributed to the growing molecular investigations on *A. baumannii* by providing evidence on the presence of multiple and multi-class carbapenemase genes and unique *bla*_OXA-51-like_ alleles among the local clinical isolates. Specifically, we reported the occurrence of cryptic Ambler class A, B, and D carbapenemase genes in some CRAb and CSAb isolates and discovered 23 novel *bla*_OXA-51-like_ alleles mostly clustering with *bla*_OXA-66_ (international clonal complex 2).

Our findings demonstrated that most of the CRAb isolates phenotypically produced carbapenemases as evident in their meropenem and imipenem resistance profiles and their capacity to degrade carbapenems in disk diffusion-based assays. Although the chosen phenotypic tests (MHT, mCIM, CIM-Tris) produced inconsistent results, all assays confirmed that carbapenemase production is more common among CRAb isolates than CSAb. There is currently no gold standard for the phenotypic detection of carbapenemase production in *A. baumannii* due to the inherently low specificity and reproducibility of the available disk diffusion-based assays [21, 22, 33, 34]. Hence, it is not surprising to see non-concordant results when comparing the phenotypic assay result to the AST profile. Another potential explanation for this non-concordance is the involvement of porin loss or efflux pump overexpression in the carbapenem-resistant phenotype [7] which is not detected in the selected disk diffusion-based assays. At least ten other potentially more accurate and rapid phenotypic tests for carbapenemase production in *A. baumannii* have been described in the last decade, but none has been accepted globally for use in the clinical setting [33]. Hence, phenotypic testing alone may not be reliable in determining actual carbapenemase production (or the propensity to produce the enzyme) in *A. baumannii*. Molecular genotyping remains to be useful in inferring carbapenemase production and activity among *A. baumannii* clinical isolates [35].

Ambler class D carbapenemases predominate in CRAb globally. As expected, all our *A. baumannii* clinical isolates possessed *bla*_OXA-51-like_ which is known to be an intrinsic carbapenemase gene in the pathogen [36]. However, recent studies revealed that *bla*_OXA-51-like_ genes can also be discovered in non-*baumannii* species of *Acinetobacter* [37], in some *Enterobacteriaceae* species [38], and in *Pseudomonas aeruginosa* [39] due to the transfer activity of the gene’s frequently linked insertion sequence, *ISAba1*. Aside from facilitating gene transfer, *ISAba1* is also associated with carbapenem resistance due to its ability to induce overexpression of the intrinsic *bla*_OXA-51-like_ genes [40]. Our findings supported this association by demonstrating that most of our CRAb isolates had the *ISAba1* upstream their *bla*_OXA-51-like_ genes. However, we also detected the *ISAba1-bla*_OXA-51-like_ complex in a few CSAb isolates, indicating that the presence of this gene complex alone is not a guaranteed predictor of carbapenem-resistant phenotype in *A. baumannii*, as also described in previous studies [41]. Exploring the genetic environment of the *ISAba1-bla*_OXA-51-like_ genes through whole genome analysis of the carbapenem-susceptible isolates could potentially explain this observed phenomenon.

Our research showed that *bla*_OXA-23-like_ genes dominated in our *A. baumannii* clinical isolates. This result corroborated with the findings of the recent genomic surveillance of *A. baumannii* in the Philippines [20] and the SENTRY study indicating *bla*_OXA-23-like_ as the most prevalent carbapenemase genes among *A. baumannii* in the Asia–Pacific region [18]. While most reports of this gene in *A. baumannii* were correlated with carbapenem resistance, recent research revealed that it can also be associated with CSAb strains as demonstrated by our findings [42]. This phenomenon can be considered a silent threat as the carbapenem-susceptible strains could serve as reservoirs of transferrable carbapenemase genes such as *bla*_OXA-23-like_ [43]. This case demonstrates the role of molecular epidemiologic research in the effective management and control of *A. baumannii*, wherein the phenotypic carbapenem susceptibility of the isolate may conceal the isolate’s potential to be a source of significant drug-resistant genes. The same situation could be true for other transferable carbapenemase genes such as *bla*_OXA-24/40-like_ and *bla*_OXA-58-like_ as these genes were also detected in our CSAb isolates, albeit only in a few strains. Notably, all our isolates harboring the *bla*_OXA-58-like_ genes have slightly higher MIC in meropenem (0.5 mg/L) compared to those without (≤ 0.25 mg/mL). This seemingly insignificant increase in MIC may be easily dismissed in the clinical laboratory but may contribute to the undetectable spread of *bla*_OXA-58-like_ genes [44]. Overall, the high occurrence of the class D *ISAba1-bla*_OXA-51-like_ and *bla*_OXA-23-like_ genes in the local isolates could be driven by high antibiotic pressure (i.e., from extensive third-generation cephalosporin and carbapenem usage) which promotes the interchange of oxacillinases between strains through mobile insertion sequences [45].

Class A *bla*_KPC_ and class B *bla*_NDM_ and *bla*_VIM_ were just recently found in *A. baumannii* and were associated with significant carbapenem resistance [8–10]. In the Philippines, *bla*_KPC-2_ was reported from one CRAb isolate through genomic surveillance [20]. In the literature, *bla*_KPC_ gene is only found in CRAb, but our findings suggested that some CSAb isolates may also harbor the gene. Similar to our observations in the *bla*_OXA-58-like_ genes, three out of five of our CSAb isolates harboring *bla*_KPC_ gene have slightly higher MIC in meropenem (0.5 mg/L). We speculate that the genetic environment of this gene in the individual *A. baumannii* strains influences the expression of the carbapenem-resistant phenotype in this species as recently discovered in genome studies [46]. Similarly, the presence of *bla*_VIM_ did not directly translate to carbapenem-resistant phenotype in some of our isolates. These findings suggest that CSAb in the clinical context may also be potent carriers of serine carbapenemases and metallo-β-lactamases that could be expressed later on as carbapenem-resistant phenotype or transferred to other Gram-negative bacteria. This phenomenon of cryptic carbapenemase genes, wherein carbapenem-susceptible pathogens do not immediately express the carbapenemase genes but may do so upon exposure to high levels of carbapenems, has been described in other recent reports [47, 48]. Similar high occurrence of cryptic carbapenemase genes in CSAb has been recently described in Indonesia [49], potentially indicating an underexplored yet rampant presence of this phenomenon in Southeast Asia. Finally, our only isolate with detected *bla*_NDM_ was reported to be CRAb, although the gene’s contribution to the carbapenem-resistant phenotype is not clear as other carbapenemase genes (*ISAba1-bla*_OXA-51-like_, *bla*_OXA-23-like_) coexist within the strain.

In general, modern strains of Gram-negative clinical isolates have multiple β-lactamase (i.e., carbapenemase) genes due to the extensive genetic transfer between species [50]. Our results showed the co-occurrence of multiple class D or class D in combination with class A or B carbapenemase genes in a few CRAb and CSAb isolates. Our study added evidence on the co-occurrence of multiple and multi-class carbapenemase genes in *A. baumannii* clinical isolates, making them potent reservoirs of various transferrable *β*-lactamase genes. This discovery has significant implications in *A. baumannii* infection management and control as the choice of antibiotics and outbreak investigations depend on the types of carbapenemase genes present in the pathogen. Evidently, recent studies showed that specific carbapenemase genes (i.e., *ISAba1-bla*_OXA-51-like_, *bla*_OXA-23-like_) are significant determinants of patient mortality and hospital outbreaks [51].

In addition to the occurrence of carbapenemase genes, we also investigated the diversity of the intrinsic *bla*_OXA-51-like_ genes among our isolates to determine locally occurring alleles and infer pathogen clonality. Since its discovery in 2005, there are already more than 370 alleles of *bla*_OXA-51-like_ genes, with the list still growing (http://www.bldb.eu/alignment.php?align=D:OXA-51-like, accessed on 5 April 2023). Our study added 23 new *bla*_OXA-51-like_ alleles from our clinical isolates, potentially indicating the unique molecular characteristics of *A. baumannii* in the locality. In addition, our SBT analysis revealed that the majority of the isolates clustered under the international clonal complexes 2 and 1 due to the presence of *bla*_OXA-66_ and *bla*_OXA-69_ alleles, respectively. This finding is supported by previous studies correlating SBT with MLST clones [15, 52, 53].

Among the new alleles reported from our study, two genes (*bla*_OXA-1064_ and *bla*_OXA1075_) showed mutations in one predicted active carbapenemase site (K217). Particularly, the lysine moiety at amino acid position 217 was hypothesized to be involved in connecting the two clefts of the active site through hydrogen bonding with serine residue in one of the protein’s loops [32]. Hence, mutations in this site could lessen the ability of the OXA-51 enzyme to hydrolyze carbapenems. This finding could potentially explain the carbapenem-susceptible phenotype of the strains where the alleles were discovered. Further enzymatic expression and kinetics study on these alleles will be needed to confirm the effect of the observed mutations on the carbapenemase activity of the enzymes. None of the new alleles had the I129L or L167V mutations predicted to increase the carbapenemase activity of OXA-51 [54].

Overall, our study presented several limitations. For instance, only a limited number of clinically significant *A. baumannii* isolates were collected and successfully maintained from the clinical laboratory (due to missed and unmaintained cultures). Despite this, we gathered representatives from both CRAb and CSAb isolates, limiting the bias of molecularly characterizing only the resistant isolates. This isolate collection strategy helped us reveal that some CSAb strains may possess cryptic carbapenemase genes which could be expressed later on (as CRAb phenotype) or transferred to other species. Meanwhile, in terms of the choice of the genes, we only used the targets commonly reported in *A. baumannii* [7] and those with available appropriate controls to minimize reporting false positives. Meanwhile, we only deduced the clonality of our strains from SBT, not MLST. Despite this, SBT using *bla*_OXA-51-like_ has been used previously to correctly infer the major clones of *A. baumannii,* corroborating with MLST findings [27–29]. Finally, this study was limited to using single gene-based analyses and genotyping experiments, not whole genome sequencing. Despite this, we ensured the validity of our results by incorporating appropriate quality check measures.

## Conclusions

Our research emphasized the value of molecular genotyping for identifying and monitoring carbapenem resistance in *A. baumannii*. The rampant spread of transferable carbapenemase genes and rapid clonal expansion of CRAb necessitate reporting of the molecular characteristics of these isolates, especially in the Philippine clinical setting which only utilizes unreliable phenotypic tests for carbapenemase detection in *A. baumannii*. In conclusion, our investigation reported the dominance of *ISAba1-bla*_OXA-51-like_ and *bla*_OXA-23-like_ genes among the clinical isolates, confirming earlier local studies. Meanwhile, we also reported the occurrence of multiple carbapenemase genes even in CSAb strains, indicating the presence of cryptic carbapenemases that could be expressed into resistant phenotype or transferred to other pathogens. Finally, our investigation discovered new *bla*_OXA-51-like_ alleles, suggesting a potentially distinct molecular epidemiology of the local isolates that calls for more surveillance. Future studies may delve into the whole genome characteristics of the *A. baumannii* isolates with interesting genotypic profiles to reveal the genetic environment of the important carbapenemase genes. Enzymatic investigations on the rare alleles may also aid in understanding the actual impact of single amino acid mutations in the activity of the intrinsic *bla*_OXA-51-like_ genes in *A. baumannii*.

## Supplementary Information

Additional file 1: Supplementary data on the methods and experimental outcomes. This additional file contains tables of the detailed characteristics of the isolates, Polymerase Chain Reaction protocols, GenBank accession numbers of the reference and original sequences used in the analysis, raw gel profiles of PCR amplicons, and complete multiple sequence alignment of the genes investigated

## Data Availability

All original DNA sequence data were deposited in the GenBank database (https://www.ncbi.nlm.nih.gov/genbank/) with accession numbers OM617740-OM617792. All other relevant data were supplied within the paper and the supplementary data.
