# Supplementary material for: Molecular genotyping reveals multiple carbapenemase genes and unique blaOXA-51-like (oxaAb) alleles among clinically isolated Acinetobacter baumannii from a Philippine tertiary hospital
Source: Trop Med Health. 2024 Sep 26;52:62. doi: 10.1186/s41182-024-00629-w (PMC11426070; doi:10.1186/s41182-024-00629-w)
Supplement: Supplementary file 1 — Additional file 1: Supplementary data on the methods and experimental outcomes. This additional file contains tables of the detailed characteristics of the isolates, Polymerase Chain Reaction protocols, GenBank accession numbers of the reference and original sequences used in the analysis, raw gel profiles of PCR amplicons, and complete multiple sequence alignment of the genes investigated [file 41182_2024_629_MOESM1_ESM.docx]

**Molecular genotyping reveals multiple carbapenemase genes and unique *bla*_OXA-51-like_ (*oxaAb)* alleles among clinically isolated *Acinetobacter baumannii* from a Philippine tertiary hospital**

**ADDITIONAL FILE 1**

**Supplementary Table 1.** Sample source and minimum inhibitory concentrations (MIC) of the *A. baumannii* clinical isolates against imipenem and meropenem.

| Isolate code | Isolation source | Imipenem MIC (mg/L) | Meropenem MIC (mg/L) |
| --- | --- | --- | --- |
| *Acinetobacter baumannii* TMC04 | Urine | ≤0.25 | ≤0.25 |
| *Acinetobacter baumannii* TMC06 | Blood | ≤0.25 | ≤0.25 |
| *Acinetobacter baumannii* TMC07 | Blood | ≥16 | ≥16 |
| *Acinetobacter baumannii* TMC08 | Sputum | ≥16 | ≥16 |
| *Acinetobacter baumannii* TMC09 | Blood | ≥16 | ≥16 |
| *Acinetobacter baumannii* TMC10 | Endotracheal aspirate | ≥16 | ≥16 |
| *Acinetobacter baumannii* TMC13 | Wound | ≥16 | ≥16 |
| *Acinetobacter baumannii* TMC16 | Sputum | ≥16 | ≥16 |
| *Acinetobacter baumannii* TMC17 | Sputum | ≥16 | ≥16 |
| *Acinetobacter baumannii* TMC19 | Blood | ≥16 | ≥16 |
| *Acinetobacter baumannii* TMC20 | Wound | ≥16 | ≥16 |
| *Acinetobacter baumannii* TMC21 | Endotracheal aspirate | ≥16 | ≥16 |
| *Acinetobacter baumannii* TMC22 | Wound | ≤0.25 | ≤0.25 |
| *Acinetobacter baumannii* TMC26 | Endotracheal aspirate | ≥16 | ≥16 |
| *Acinetobacter baumannii* TMC27 | Endotracheal aspirate | ≥16 | ≥16 |
| *Acinetobacter baumannii* TMC28 | Blood | ≤0.25 | ≤0.25 |
| *Acinetobacter baumannii* TMC29 | Sputum | ≤0.25 | ≤0.25 |
| *Acinetobacter baumannii* TMC31 | Endotracheal aspirate | ≤0.25 | 0.5 |
| *Acinetobacter baumannii* TMC32 | Sputum | ≤0.25 | ≤0.25 |
| *Acinetobacter baumannii* TMC33 | Sputum | ≤0.25 | 0.5 |
| *Acinetobacter baumannii* TMC34 | Sputum | ≤0.25 | ≤0.25 |
| *Acinetobacter baumannii* TMC36 | Sputum | ≤0.25 | ≤0.25 |
| *Acinetobacter baumannii* TMC39 | Sputum | ≤0.25 | ≤0.25 |
| *Acinetobacter baumannii* TMC40 | Blood | ≤0.25 | ≤0.25 |
| *Acinetobacter baumannii* TMC42 | Urine | ≤0.25 | 0.5 |
| *Acinetobacter baumannii* TMC43 | Sputum | ≤0.25 | 0.5 |
| *Acinetobacter baumannii* TMC48 | Urine | ≤0.25 | 0.5 |
| *Acinetobacter baumannii* TMC51 | Abscess | ≤0.25 | ≤0.25 |
| *Acinetobacter baumannii* TMC54 | Endotracheal aspirate | ≤0.25 | ≤0.25 |
| *Acinetobacter baumannii* TMC55 | Endotracheal aspirate | ≤0.25 | 0.5 |
| *Acinetobacter baumannii* TMC56 | Blood | ≤0.25 | ≤0.25 |
| *Acinetobacter baumannii* TMC58 | Sputum | ≤0.25 | 0.5 |
| *Acinetobacter baumannii* TMC60 | Wound | ≤0.25 | 1 |
| *Acinetobacter baumannii* TMC63 | Endotracheal aspirate | ≥16 | ≥16 |
| *Acinetobacter baumannii* TMC64 | Urine | ≤0.25 | ≤0.25 |
| *Acinetobacter baumannii* TMC67 | Endotracheal aspirate | ≤0.25 | 0.5 |
| *Acinetobacter baumannii* TMC68 | Endotracheal aspirate | ≤0.25 | 0.5 |
| *Acinetobacter baumannii* TMC69 | Wound | ≥16 | ≥16 |
| *Acinetobacter baumannii* TMC73 | Blood | ≥16 | ≥16 |
| *Acinetobacter baumannii* TMC74 | Blood | ≥16 | ≥16 |
| *Acinetobacter baumannii* TMC75 | Sputum | ≥16 | ≥16 |
| *Acinetobacter baumannii* TMC76 | Urine | ≥16 | ≥16 |
| *Acinetobacter baumannii* TMC77 | Endotracheal aspirate | ≥16 | ≥16 |
| *Acinetobacter baumannii* TMC78 | Endotracheal aspirate | ≥16 | ≥16 |
| *Acinetobacter baumannii* TMC79 | Endotracheal aspirate | ≥16 | ≥16 |
| *Acinetobacter baumannii* TMC80 | Blood | ≤0.25 | ≤0.25 |
| *Acinetobacter baumannii* TMC81 | Wound | ≤0.25 | ≤0.25 |
| *Acinetobacter baumannii* TMC82 | Urine | ≥16 | ≥16 |
| *Acinetobacter baumannii* TMC83 | Blood | ≥16 | ≥16 |
| *Acinetobacter baumannii* TMC84 | Endotracheal aspirate | ≥16 | ≥16 |
| *Acinetobacter baumannii* TMC85 | Endotracheal aspirate | ≥16 | ≥16 |
| *Acinetobacter baumannii* TMC86 | Sputum | ≤0.25 | ≤0.25 |

**Supplementary Table 2.** List of primers, polymerase chain reaction (PCR) parameters, and controls used for the detection of the target carbapenemase genes.

| Primer names* | Primer sequence (5’-3’) | Expected amplicon size (bp) | AT^#^ (°C) | Positive control | Ref^§^ |
| --- | --- | --- | --- | --- | --- |
| KPC-F | ATGTCACTGTATCGCCGTCT | 882 | 63 | *Klebsiella pneumoniae* ATCC BAA-1705™ | [1] |
| KPC-R | TTACTGCCCGTTGACGCCC |  |  |  |  |
| NDM-F | GGTTTGGCGATCTGGTTTTC | 621 | 60 | *Klebsiella pneumoniae* ATCC BAA-2146™ | [2] |
| NDM-R | CGGAATGGCTCATCACGATC |  |  |  |  |
| IMP-F | GGAATAGAGTGGCTTAATTCTC | 233 | 52 | *Escherichia coli* NCTC 13476™ | [3] |
| IMP-R | GGTTTAACAAAACAACCACC |  |  |  |  |
| VIM-F | ATGTTAAAAGTTATTAGTAGTTTATTG | 801 | 57 | *Klebsiella pneumoniae* NCTC 13440™ | [2] |
| VIM-R | CTACTCGGCGACTGAGC |  |  |  |  |
| OXA-23-F | CTATTTTTGTCGTGTACAGAGC | 1008 | 62 | *Acinetobacter baumannii* ATCC BAA-1605™ | [4] |
| OXA-23-R | GGATCACAACAACTAAAAGCACTG |  |  |  |  |
| OXA-24/40-F | GGTTAGTTGGCC CCCTTAAA | 249 | 53 |  | [5] |
| OXA-24/40-R | AGTTGAGCGAAA AGGGGATT |  |  |  |  |
| OXA-48-F | TTGGTGGCATCGATTATCGG | 744 | 52 | *Klebsiella penumoniae* NCTC 13442™ | [6] |
| OXA-48-R | GAGCACTTCTTTTGTGATGGC |  |  |  |  |
| OXA-51-F | CCATGGCAATGAACATTAAAGCACTCTTAC | 825 | 51 | *Acinetobacter baumannii* ATCC BAA-1605™ | [7] |
| OXA-51-R | CTATAAAATACCTAATTGTTCT |  |  |  |  |
| OXA-58-F | AAGTATTGGGGCTTGTGCTG | 599 | 58 | *Acinetobacter baumannii* strain ADD | [8] |
| OXA-58-R | CCCCTCTGCGCTCTACATAC |  |  |  |  |
| ISAba1-F | CACGAATGCAGAAGTTG | 1,200 | 57 | *Acinetobacter baumannii* strain RMD | [9] |
| OXA-51-F | CCATGGCAATGAACATTAAAGCACTCTTAC |  |  |  |  |

*F- forward primer, R- reverse primer; KPC- *Klebsiella pneumoniae* carbapenemase, NDM- New Delhi Metallo-β-lactamase, IMP- Imipenemase, VIM- Verona Integron-associated Metallo-β-lactamase, OXA- Oxacillinase, ISAba1- *Acinetobacter baumannii* Insertion Sequence 1

^#^Annealing temperature in degrees Celsius

^§^References:

[1] Mohammed Y, Zailani SB, Onipede AO. Characterization of KPC , NDM and VIM type carbapenem resistance *Enterobacteriaceae* from North Eastern Nigeria. J Biosci Med 2015; 3:100–107.

[2] Juan C, Beceiro A, Gutiérrez O, et al. Characterization of the new metallo-beta-lactamase VIM-13 and its integron-borne gene from a *Pseudomonas aeruginosa* clinical isolate in Spain. Antimicrob Agents Chemother 2008;52(10):3589-3596.

[3] Poirel L, Walsh TR, Cuvillier V, Nordmann P. Multiplex PCR for detection of acquired carbapenemase genes. Diagn Microbiol Infect Dis 2011;70(1):119-123.

[4] Yang Z, Wang P, Song P, Li X. Carbapenemase OXA-423: A Novel OXA-23 Variant in *Acinetobacter baumannii*. Infect Drug Resist 2020;13:4069-4075.

[5] Woodford N, Ellington MJ, Coelho JM, et al. Multiplex PCR for genes encoding prevalent OXA carbapenemases in *Acinetobacter* spp. Int J Antimicrob Agents 2006;27(4):351-353.

[6] Salloum NA, Kissoyan KA, Fadlallah S, et al. Assessment of combination therapy in BALB/c mice injected with carbapenem-resistant *Enterobacteriaceae* strains. Front Microbiol 2015;6:999.

[7] Hu WS, Yao SM, Fung CP, Hsieh YP, Liu CP, Lin JF. An OXA-66/OXA-51-like carbapenemase and possibly an efflux pump are associated with resistance to imipenem in *Acinetobacter baumannii*. Antimicrob Agents Chemother 2007;51(11):3844-3852.

[8] Sohrabi N, Farajnia S, Akhi MT, et al. Prevalence of OXA-type β-lactamases among *Acinetobacter baumannii* isolates from Northwest of Iran. Microb Drug Resist 2012;18(4):385-389.

[9] Segal H, Garny S, Elisha G. Is *ISAba-1* customized for *Acinetobacter*? FEMS Microbiol Let 2005; 243(2): 425-429.

**Supplementary Table 3.** Genbank (https://www.ncbi.nlm.nih.gov/genbank/) accession numbers of the 87 reference *bla_OXA-51-like_* genes used for the construction of the phylogenetic tree in Figure 2.

| *bla_OXA-51-like_* allele | GenBank accession number |  | *bla_OXA-51-like_* allele | GenBank accession number |
| --- | --- | --- | --- | --- |
| OXA-51 | DQ385606 |  | OXA-442 | KP844571 |
| OXA-64 | AY750907 |  | OXA-480 | KR872296 |
| OXA-65 | AY750908 |  | OXA-508 | KU596972 |
| OXA-66 | AY750909 |  | OXA-509 | KU692025 |
| OXA-68 | AY750910 |  | OXA-510 | JX865393 |
| OXA-71 | AY859528 |  | OXA-511 | KU710720 |
| OXA-76 | AY949203 |  | OXA-521 | KX462701 |
| OXA-78 | AY862132 |  | OXA-523 | KX599401 |
| OXA-79 | EU019534 |  | OXA-524 | KX599402 |
| OXA-80 | EU019535 |  | OXA-529 | KX599405 |
| OXA-82 | DQ987479 |  | OXA-536 | KX859240 |
| OXA-83 | DQ309277 |  | OXA-545 | KY674541 |
| OXA-88 | DQ392963 |  | OXA-555 | KY126224 |
| OXA-89 | DQ445683 |  | OXA-556 | KY126231 |
| OXA-90 | EU547443 |  | OXA-558 | KY126234 |
| OXA-91 | DQ519086 |  | OXA-654 | MH337646 |
| OXA-98 | EU255288 |  | OXA-680 | MH878938 |
| OXA-109 | EF650035 |  | OXA-687 | MK011137 |
| OXA-117 | GQ423625 |  | OXA-693 | MK011143 |
| OXA-121 | EU255290 |  | OXA-694 | MK011144 |
| OXA-122 | EU255291 |  | OXA-699 | MK011149 |
| OXA-127 | EU255296 |  | OXA-701 | MK011151 |
| OXA-128 | EU375515 |  | OXA-708 | MK011166 |
| OXA-132 | EU547447 |  | OXA-711 | MK011169 |
| OXA-138 | EU670845 |  | OXA-717 | MK011175 |
| OXA-144 | FJ872530 |  | OXA-733 | MH780076 |
| OXA-150 | GQ853681 |  | OXA-738 | MK105925 |
| OXA-174 | HM113560 |  | OXA-740 | MK105927 |
| OXA-200 | HQ734811 |  | OXA-742 | MK105929 |
| OXA-202 | HQ734813 |  | OXA-756 | MK105945 |
| OXA206 | AB634250 |  | OXA-757 | MK105946 |
| OXA-217 | JN603240 |  | OXA-766 | MK105955 |
| OXA-219 | JN215211 |  | OXA-831 | MK933721 |
| OXA-234 | NG_050607 |  | OXA-857 | MN476894 |
| OXA-254 | AB781687 |  | OXA-869 | MN476908 |
| OXA-312 | KF057029 |  | OXA-870 | MN476909 |
| OXA-313 | KF057030 |  | OXA-882 | MN476922 |
| OXA-336 | KF048907 |  | OXA-889 | MN476929 |
| OXA-342 | KF048915 |  | OXA-892 | MN476932 |
| OXA-343 | KF048916 |  | OXA-916 | MT437251 |
| OXA-374 | KF986255 |  | OXA-954 | MW805236 |
| OXA-377 | KF986258 |  | OXA-970 | MZ265722 |
| OXA-426 | KM588354 |  | OXA-1013 | MZ424303 |
| OXA-431 | KM979378 |  |  |  |

**Supplementary Table 4.** Genbank (https://www.ncbi.nlm.nih.gov/genbank/) accession numbers of the 52 *bla_OXA-51-like_* gene sequences annotated in the study.

| Sequence Name | GenBank ID |
| --- | --- |
| *Acinetobacter baumannii* TMC09 *bla*_OXA-66_ | OM617767 |
| *Acinetobacter baumannii* TMC16 *bla*_OXA-66_ | OM617768 |
| *Acinetobacter baumannii* TMC17 *bla*_OXA-66_ | OM617769 |
| *Acinetobacter baumannii* TMC56 *bla*_OXA-66_ | OM617770 |
| *Acinetobacter baumannii* TMC80 *bla*_OXA-66_ | OM617771 |
| *Acinetobacter baumannii* TMC82 *bla*_OXA-66_ | OM617772 |
| *Acinetobacter baumannii* TMC83 *bla*_OXA-66_ | OM617773 |
| *Acinetobacter baumannii* TMC85 *bla*_OXA-66_ | OM617774 |
| *Acinetobacter baumannii* TMC20 *bla*_OXA-68_ | OM617775 |
| *Acinetobacter baumannii* TMC54 *bla*_OXA-70_ | OM617777 |
| *Acinetobacter baumannii* TMC22 *bla*_OXA-90_ | OM617778 |
| *Acinetobacter baumannii* TMC86 *bla*_OXA-98_ | OM617779 |
| *Acinetobacter baumannii* TMC42 *bla*_OXA-100_ | OM617780 |
| *Acinetobacter baumannii* TMC28 *bla*_OXA-120_ | OM617781 |
| *Acinetobacter baumannii* TMC08 *bla*_OXA-342_ | OM617782 |
| *Acinetobacter baumannii* TMC10 *bla*_OXA-508_ | OM617783 |
| *Acinetobacter baumannii* TMC13 *bla*_OXA-508_ | OM617784 |
| *Acinetobacter baumannii* TMC26 *bla*_OXA-508_ | OM617785 |
| *Acinetobacter baumannii* TMC63 *bla*_OXA-508_ | OM617786 |
| *Acinetobacter baumannii* TMC69 *bla*_OXA-508_ | OM617787 |
| *Acinetobacter baumannii* TMC73 *bla*_OXA-508_ | OM617788 |
| *Acinetobacter baumannii* TMC74 *bla*_OXA-508_ | OM617789 |
| *Acinetobacter baumannii* TMC76 *bla*_OXA-508_ | OM617790 |
| *Acinetobacter baumannii* TMC78 *bla*_OXA-508_ | OM617791 |
| *Acinetobacter baumannii* TMC79 *bla*_OXA-508_ | OM617792 |
| *Acinetobacter baumannii* TMC07 *bla*_OXA-1058_ | OM617740 |
| *Acinetobacter baumannii* TMC21 *bla*_OXA-1059_ | OM617741 |
| *Acinetobacter baumannii* TMC51 *bla*_OXA-1060_ | OM617742 |
| *Acinetobacter baumannii* TMC75 *bla*_OXA-1061_ | OM617743 |
| *Acinetobacter baumannii* TMC19 *bla*_OXA-1062_ | OM617744 |
| *Acinetobacter baumannii* TMC27 *bla*_OXA-1063_ | OM617745 |
| *Acinetobacter baumannii* TMC84 *bla*_OXA-1064_ | OM617746 |
| *Acinetobacter baumannii* TMC48 *bla*_OXA-1065_ | OM617747 |
| *Acinetobacter baumannii* TMC60 *bla*_OXA-1065_ | OM617748 |
| *Acinetobacter baumannii* TMC04 *bla*_OXA-1066_ | OM617749 |
| *Acinetobacter baumannii* TMC18 *bla*_OXA-1066_ | OM617750 |
| *Acinetobacter baumannii* TMC29 *bla*_OXA-1066_ | OM617751 |
| *Acinetobacter baumannii* TMC31 *bla*_OXA-1067_ | OM617752 |
| *Acinetobacter baumannii* TMC58 *bla*_OXA-1067_ | OM617753 |
| *Acinetobacter baumannii* TMC68 *bla*_OXA-1068_ | OM617754 |
| *Acinetobacter baumannii* TMC64 *bla*_OXA-1069_ | OM617755 |
| *Acinetobacter baumannii* TMC67 *bla*_OXA-1070_ | OM617756 |
| *Acinetobacter baumannii* TMC36 *bla*_OXA-1071_ | OM617757 |
| *Acinetobacter baumannii* TMC39 *bla*_OXA-1072_ | OM617758 |
| *Acinetobacter baumannii* TMC43 *bla*_OXA-1073_ | OM617759 |
| *Acinetobacter baumannii* TMC33 *bla*_OXA-1074_ | OM617760 |
| *Acinetobacter baumannii* TMC06 *bla*_OXA-1075_ | OM617761 |
| *Acinetobacter baumannii* TMC81 *bla*_OXA-1076_ | OM617762 |
| *Acinetobacter baumannii* TMC55 *bla*_OXA-1077_ | OM617763 |
| *Acinetobacter baumannii* TMC40 *bla*_OXA-1078_ | OM617764 |
| *Acinetobacter baumannii* TMC34 *bla*_OXA-1079_ | OM617765 |
| *Acinetobacter baumannii* TMC32 *bla*_OXA-1080_ | OM617766 |

*bla*_KPC_


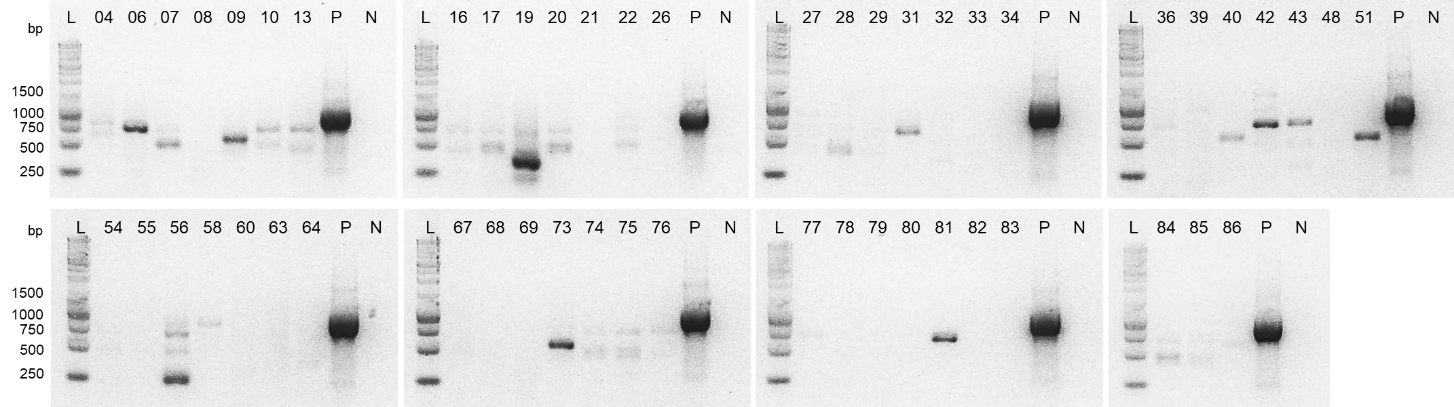


*bla*_NDM_


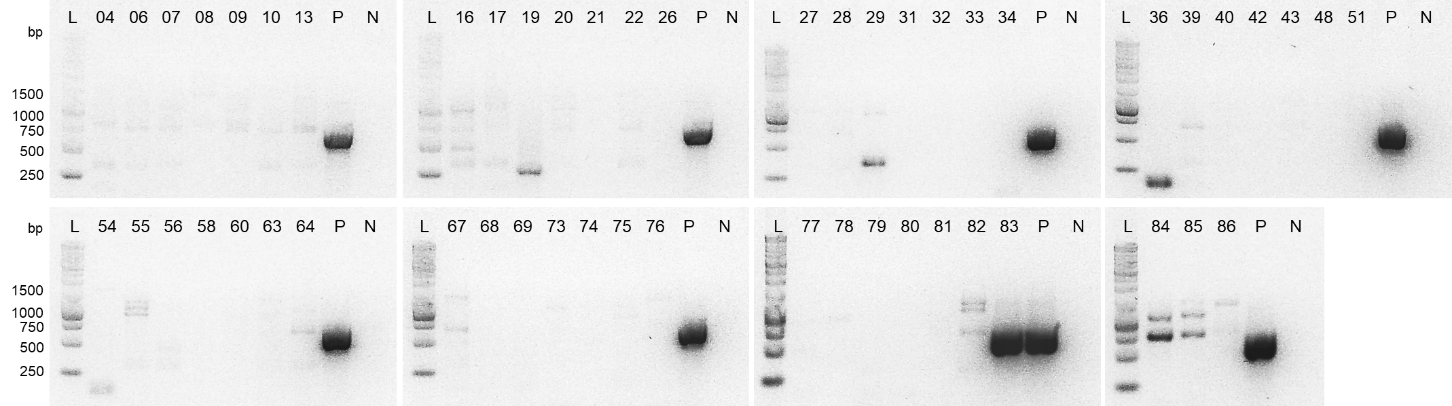


*bla*_IMP_


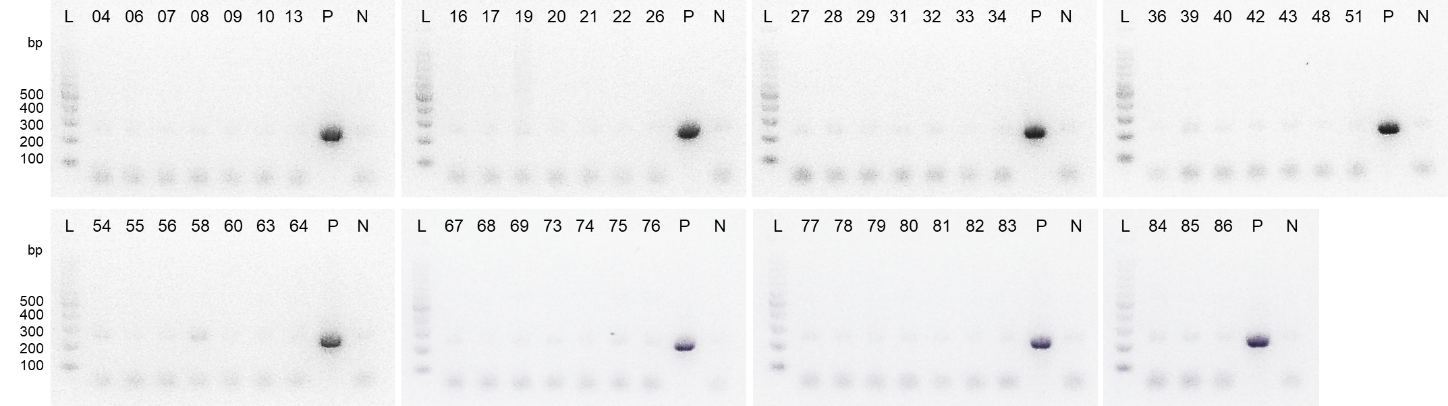


*bla*_VIM_


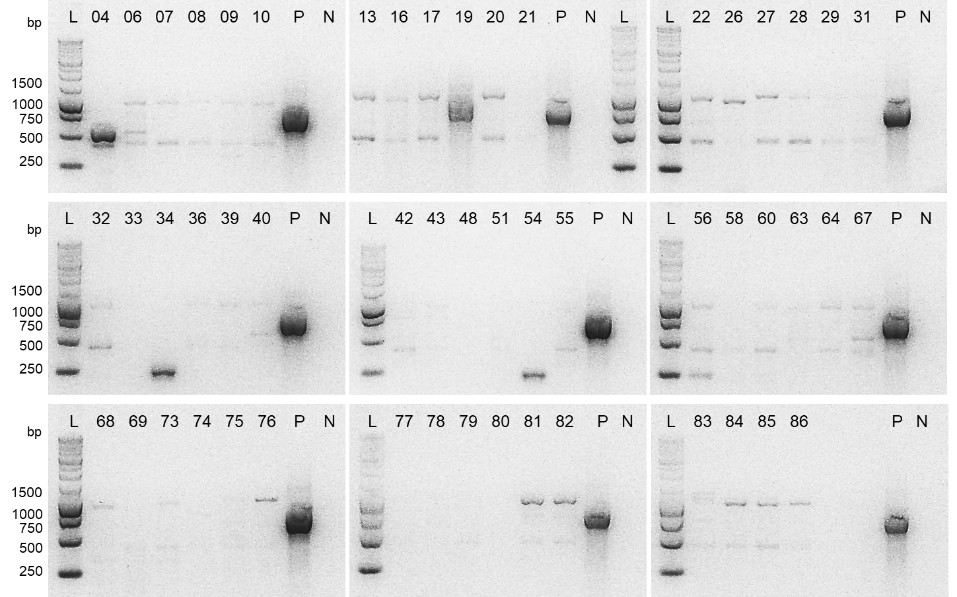


*bla*_OXA-23-like_


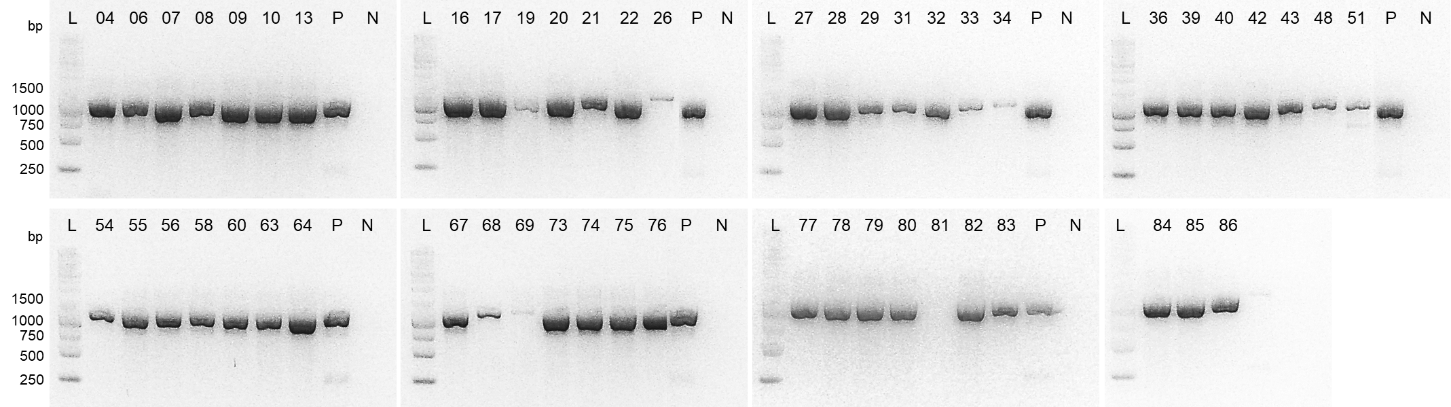


*bla*_OXA-24/40-like_


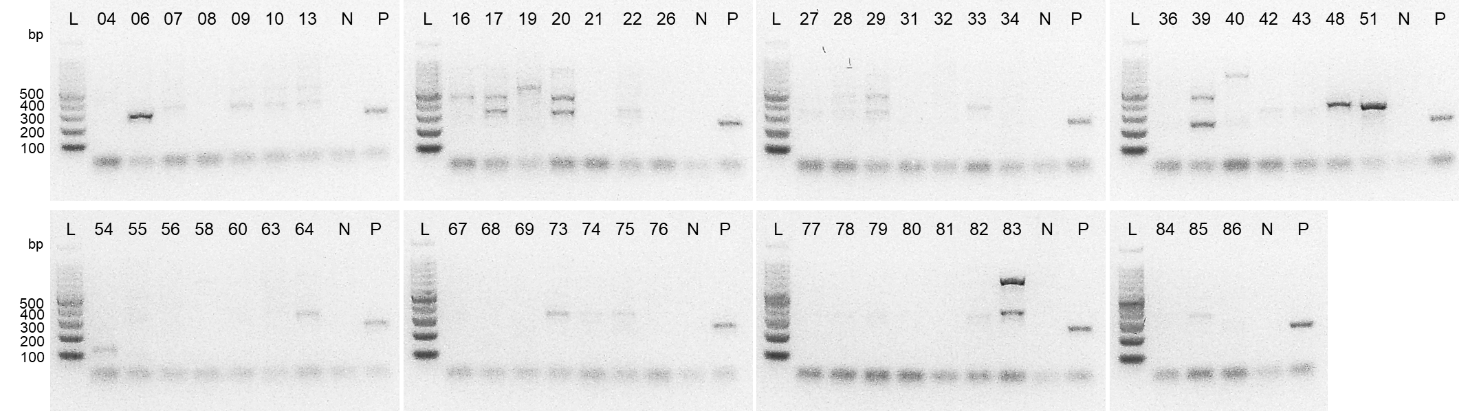


*bla*_OXA-48-like_


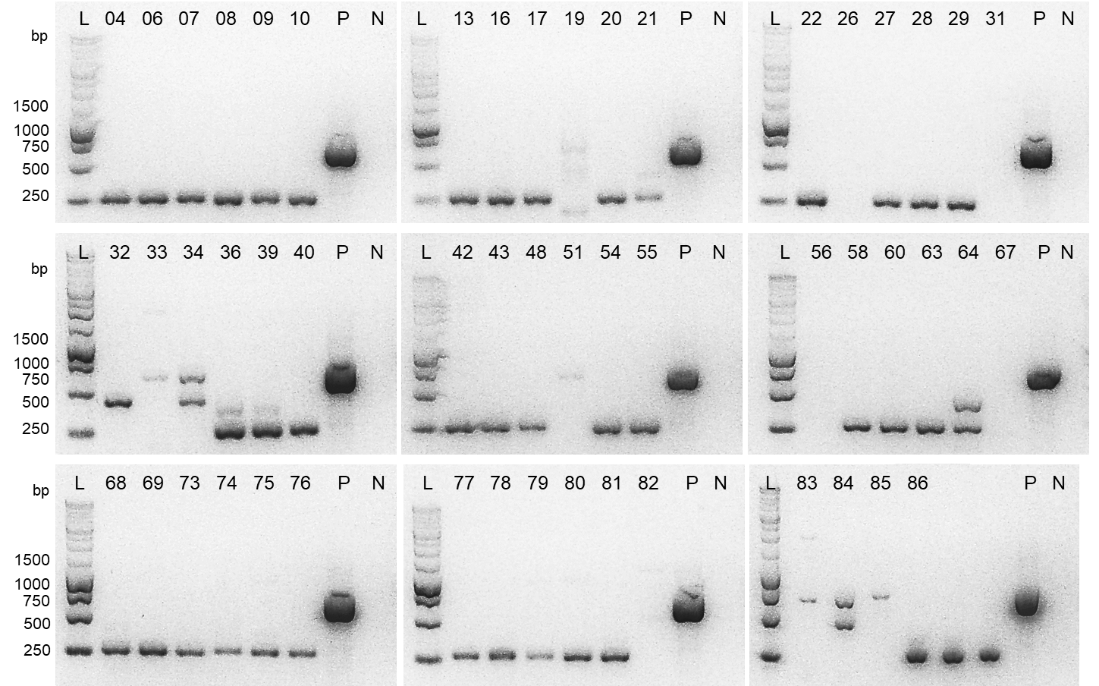


*bla*_OXA-51-like_


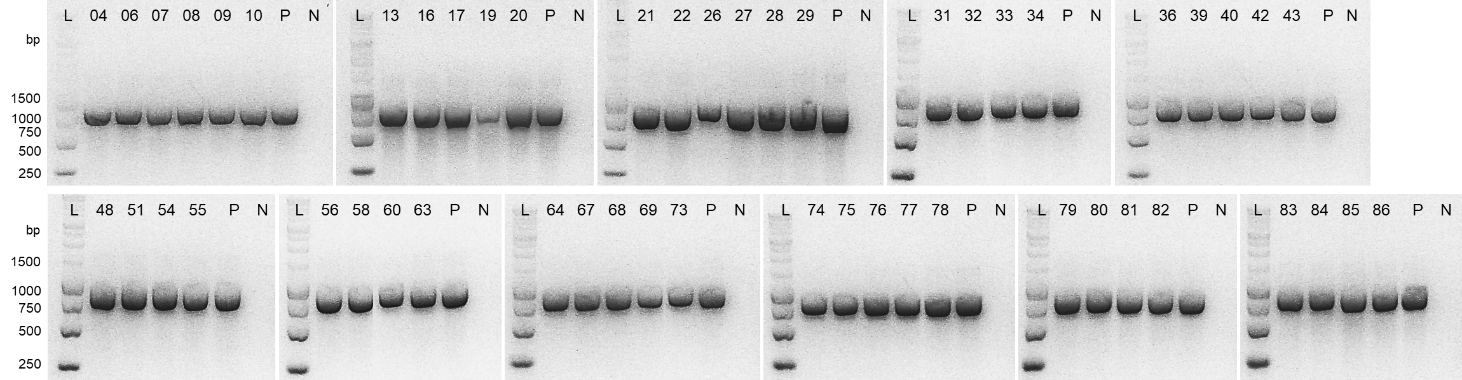


*bla*_OXA-58-like_


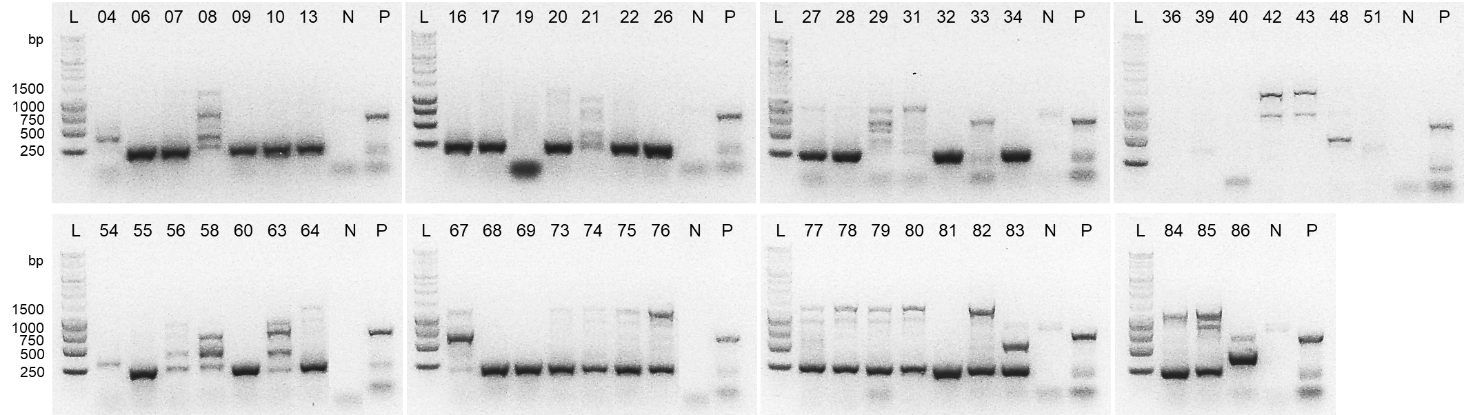


*ISAba1-bla*_OXA-51-like_


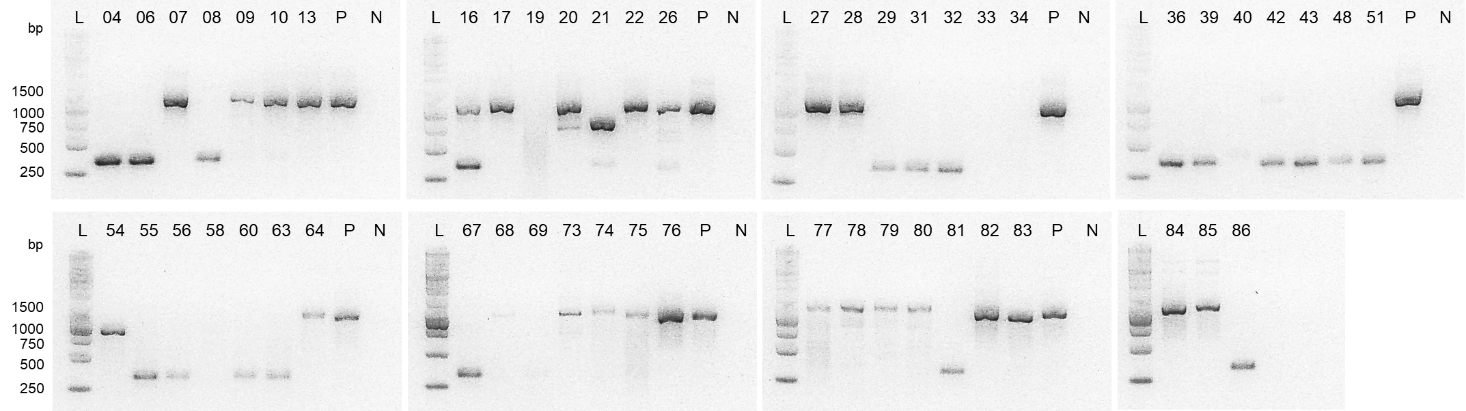


**Supplementary Figure 1.** Raw gel electrophoresis profiles of the *A. baumannii* clinical isolates (lanes referring to isolate codes) after PCR amplification targeting selected carbapenemase genes. L refers to the DNA ladder, P to the positive control (see Table S1), and N to the negative control (*Staphylococcus aureus* ATCC 29213™). The expected amplicon size for each target gene are: 882bp (*bla*_KPC_), 621bp (*bla*_NDM_), 233bp (*bla*_IMP_), 801bp (*bla*_VIM_), 1.8kbp (*bla*_OXA-23-like_), 249bp (*bla*_OXA-24/40-like_), 744bp (*bla*_OXA-48-like_), 825bp (*bla*_OXA-51-like_), 599bp (*bla*_OXA-58-like_), and 1.2kbp (*ISAba1-bla*_OXA-51-like_). Gel images were taken as inverted color images to visualize faint bands. Only the isolates with matching amplicon profiles with the positive control were interpreted as positive for the gene target. Non-specific amplicons were not considered. Gel images were compiled and annotated in Microsoft Powerpoint Program (Microsoft, USA).

....|....| ....|....| ....|....| ....|....| ....|....| ....|....| ....|....|

10 20 30 40 50 60 70

**OXA-66**  MNIKALLLIT SAIFISACSP YIVTANPNHS ASKSDVKAEK IKNLFNEAHT TGVLVIQQGQ TQQSYGNDLA

**OXA-1058**  MNSKALLLIT SAIFISACSP YIVTANPNHS ASKSDVKAEK IKNLFNEAHT TGVLVIQQGQ TQQSYGNDLA

**OXA-1059**  MNIQTLLLIT SAIFISACSP YIVTANPNHS ASKSDVKAEK IKNLFNEAHT TGVLVIQQGQ TQQSYGNDLA

**OXA-1060**  MNIKTLLLIT SAIFISACSP YIVSANPNHS ASKSDVKAEK IKNLFNEAHT TGVLVIQQGQ TQQSYGNDLA

**OXA-1061**  MNIKTLLLIT SAIFISACSP YIVTANPNHS ASKSDVKAEK IKNLFNEAHT TGVLVIQQGQ TQQSYGNDLA

**OXA-1062**  MNIKSLLLIT SAIFISACSP YIVTANPNHS ASKSDVKAEK IKNLFNEAHT TGVLVIQQGQ TQQSYGNDLA

**OXA-1063**  MNIKTLLLIT SAIFISACSP YIVTANPNHS ASKSDVKAEK IKNLFNEAHT TGVLVIQQGQ TQQSYGNDLA

**OXA-1064**  MNIKTLLLIT SAIFISACSP YIVTANPNHS ASKSDVKAEK IKNLFNEAHT TGVLVIQQGQ TQQSYGNDLA

**OXA-1065**  MNIKTLLLIT SAIFISACSP YIVTANPNHS ASKSDEKAEK IKNLFNEAHT TGVLVIQQGQ TQQSYGNDLA

**OXA-1066**  MNIKTLLLIT SAIFISACSP YIVTANPNHS ASKSDEKAEK IKNLFNEAHT TGVLVIQQGQ TQQSYGNDLA

**OXA-1067**  MNIKTLLLIT SAIFISACSP YIVSANPNHS ASKSDEKAEK IKNLFNEAHT TGVLVIQQGQ TQQSYGNDLA

**OXA-1068**  MNIISILLIT SAIFISACSP YIVSANPNHS ASKSDEKAEK IKNLFNEAHT TGVLVIQQGQ TQQSYGNDLA

**OXA-1069**  MNIKTLLLIT SAIFISACSP YIVSANPNHS ASKSDEKAEK IKNLFNEAHT TGVLVIQQGQ TQQSYGNDLA

**OXA-1070**  MNIKALLLIT SAIFISACSP YIVSANPNHS ASKSDEKAEK IKNLFNEAHT TGVLVIQQGQ TQQSYGNDLA

**OXA-1071**  MNIKALLLIT SAIFISACSP YIVTANPNHS ASKSDEKAEK IKNLFNEVHT TGVLVIQQGQ TQQSYGNDLA

**OXA-1072**  MNIKALLLIT SAIFISACSP YIVTANPNHS ASKSDEKAEK IKNLFNEVHT TGVLVIQQGQ TQQSYGNDLA

**OXA-1073**  MNIKTLLLIT SAIFISACSP YIVTANPNHS ASKSDEKAEK IKNLFNEAHT TGVLVIQQGQ TQQSYGNDLA

**OXA-1074**  MNIKALLLIT SAIFISACSP YIVTANPNHS ASKSDDKAEK IKNLFNEAHT TGVLVIHQGQ TQQSYGNDLA

**OXA-1075**  MNIKTLLLIT SAIFISACSP YIVSANPNHS ASKSDEKAEK IKNLFNEAHT TGVLVIQQGQ TQQSYGNDLA

**OXA-1076**  MNIKALLLIT SAIFISACSP YIVTTNPNHS ASKSDEKAEK IKNLFNEAHT TGVLVIQQGQ TQQSYGNDLA

**OXA-1077**  MNIKTLLLIT SAIFISACSP YIVSANPNHS ASKSDEKAEK IKNLFNEAHT TGVLVIQQGQ TQQSYGNDLA

**OXA-1078**  MNIKTLLLIT STIFISACSP YIVTANPNHS TSKSDEKAEK IKNLFNEAHT TGVLVIQQGQ TQQSYGNDLA

**OXA-1079**  MNIKALLLIT SAIFISACSP YIVTANPNHS ASKSDDKAEK IKNLFNEAHT TGVLVIHQGQ TQQSYGNDLA

**OXA-1080**  MNIKALLLIT SAIFISACSP YIVTANPNHS ASKSDVKAEK IKNLFNEAHT TGVLVIQQGQ TQQSYGNDLA

....|....| ....|....| ....|....| ....|....| ....|....| ....|....| ....|....|

80 90 100 110 120 130 140

**OXA-66**  RASTEYVPAS TFKMLNALIG LEHHKATTTE VFKWDGKKRL FPEWEKDMTL GDAMKASAIP VYQDLARRIG

**OXA-1058**  RASTEYVPAS TFKMLNALIG LEHHKATTTE VFKWDGKKRL FPEWEKDMTL GDAMKASAIP VYQDLARRIG

**OXA-1059**  RASTEYVPAS TFKMLNALIG LEHHKATTTE VFKWDGKKRL FPEWEKDMTL GDAMKASAIP VYQDLARRIG

**OXA-1060**  RASTEYVPAS TFKMLNALIG LEHHKATTTE VFKWDGQKRL FPEWEKDMTL GDAMKASAIP VYQDLARRIG

**OXA-1061**  RASTEYVPAS TFKMLNALIG LEHHKATTTE VFKWDGKKRL FPEWEKDMTL GDAMKASAIP VYQDLARRIG

**OXA-1062**  RASTEYVPAS TFKMLNALIG LEHHKATTTE VFKWDGKKRL FPEWEKDMTL GDAMKASAIP VYQDLARRIG

**OXA-1063**  RASTEYVPAS TFKMLNALIG LEHHKATTTE VFKWDGKKRL FPEWEKDMTL GDAMKASAIP VYQDLARRIG

**OXA-1064**  RASTEYVPAS TFKMLNALIG LEHHKATTTE VFKWDGKKRL FPEWEKDMTL GDAMKASAIP VYQDLARRIG

**OXA-1065**  RASTEYVPAS TFKMLNALIG LEHHKATTTE VFKWDGKKRL FPEWEKDMTL GDAMKASAIP VYQDLARRIG

**OXA-1066**  RASTEYVPAS TFKMLNALIG LEHHKATTTE VFKWDGKKRL FPEWEKDMTL GDAMKASAIP VYQDLARRIG

**OXA-1067**  RASTEYVPAS TFKMLNALIG LEHHKATTTE VFKWDGQKRL FPEWEKNMTL GDAMKASAIP VYQDLARRIG

**OXA-1068**  RASTEYVPAS TFKMLNALIG LEHHKATTTE VFKWDGQKRL FPEWEKNMTL GDAMKASAIP VYQDLARRIG

**OXA-1069**  RASTEYVPAS TFKMLNALIG LEHHKATTTE VFKWGGQKRL FPEWEKNMTL GDAMKASAIP VYQDLARRIG

**OXA-1070**  RASTEYVPAS TFKMLNALIG LEHHKATTTE VFKWDGQKRL FPEWEKDMTL GDAMKASAIP VYQDLARRIG

**OXA-1071**  RASTEYVPAS TFKMLNALIG LEHHKATTTE VFKWDGQKRL FPEWEKDMTL GDAMKASAIP VYQDLARRIG

**OXA-1072**  RASTEYVPAS TFKMLNALIG LEHHKATTTE VFKWDGQKRL FPEWEKDMTL GDAMKASAIP VYQDLARRIG

**OXA-1073**  RASTEYVPAS TFKMLNALIG LEHHKATTTE VFKWDGQKRL FPEWEKDMTL GDAMKASAIP VYQDLARRIG

**OXA-1074**  RASTEYVPAS TFKMLNALIG LEHHKATTTE VFKWDGQKRL FPEWEKDMTL GDAMKASAIP VYQDLARRIG

**OXA-1075**  RASTEYVPAS TFKMLNALIG LEHHKATTTE VFKWDGQKRL FPEWEKNMTL GDAMKASAIP VYQDLARRIG

**OXA-1076**  RASTEYVPAS TFKMLNALIG LEHHKTTTTE VFKWDGQKRL FPEWEKDMTL GDAMKASAIP VYQDLARRIG

**OXA-1077**  RASTEYVPAS TFKMLNALIG LEHHKATTTE VFKWDGQKRL FPEWEKNMTL GDAMKASAIP VYQDLARRIG

**OXA-1078**  RASTEYVPAS TFKMLNALIG LEHHKATTTE IFKWDGQKRL FPEWEKDMTL GDAMKASAIP VYQDLARRIG

**OXA-1079**  RASTEYVPAS TFKMLNALIG LEHHKATTTE IFKWDGQKRL FPEWEKDMTL GDAMKASAIP VYQDLARRIG

**OXA-1080**  RASTEYVPAS TFKMLNALIG LEHHKATTTE VFKWDGKKRL FPEWEKDMTL GDAMKASAIP VYQDLARRIG

....|....| ....|....| ....|....| ....|....| ....|....| ....|....| ....|....|

150 160 170 180 190 200 210

**OXA-66**  LELMSKEVKR VGYGNADIGT QVDNFWLVGP LKITPQQEAQ FAYKLANKTL PFSQKVQDEV QSMLFIEEKN

**OXA-1058**  LELMSKEVKR VGYGNADIGT QVDNFWLVGP LKITPQQEAQ FAYKLANKTL PFSQKVQDEV QSMLFIEEKN

**OXA-1059**  LELMSKEVKR VGYGNADIGT QVDNFWLVGP LKITPQQEAQ FAYKLANKTL PFSQKVQDEV QSMLFIEEKN

**OXA-1060**  LELMSKEVKR VGYGNADIGT QVDNFWLVGP LKITPQQEAQ FAYKLANKTL PFSQKVQDEV QSMLFIEEKN

**OXA-1061**  LELMSKEVKR VGYGNADIGT QVDNFWLVGP LKITPQQEAQ FAYKLANKTL PFSQKVQDEV QSMLFIEEKN

**OXA-1062**  LELMSKEVKR VGYGNADIGT QVDNFWLVGP LKITPQQEAQ FAYKLANKTL PFSQKVQDEV QSMLFIEEKN

**OXA-1063**  LELMSKEVKR VGYGNADIGT QVDNFWLVGP LKITPQQEAQ FAYKLANKTL PFSQKVQDEV QSMLFIEEKN

**OXA-1064**  LELMSKEVKR VGYGNADIGT QVDNFWLVGP LKITPQQEAQ FAYKLANKTL PFSQKVQDEV QSMLFIEEKN

**OXA-1065**  LELMSKEVKR VGYGNADIGT QVDNFWLVGP LKITPQQEAQ FAYKLANKTL PFSQKVQDEV QSMLFIEEKN

**OXA-1066**  LELMSKEVKR VGYGNADIGT QVDNFWLVGP LKITPQQEAQ FAYKLANKTL PFSQKVQDEV QSMLFIEEKN

**OXA-1067**  LELMSNEVKR VGYGNADIGT QVDNFWLVGP LKITPQQEAQ FAYKLANKTL PFSQEVQDEV QSMLFIEEKN

**OXA-1068**  LELMSNEVKR VGYGNADIGT QVDNFWLVGP LKITPQQEAQ FAYKLANKTL PFSQEVQDEV QSMLFIEEKN

**OXA-1069**  LELMSNEVKR VGYGNADIGT QVDNFWLVGP LKITPQQEAQ FAYKLANKTL PFSQKVQDEV QSMLFIEEKN

**OXA-1070**  LELMSKEVKR VGYGNADIGT QVDNFWLVGP LKITPQQEAQ FAYKLANKTL PFSQKVQDEV QSMLFIEEKN

**OXA-1071**  LELMSKEVKR VGYGNADIGT QVDNFWLVGP LKITPQQEAQ FAYKLANKTL PFSPKVQDEV QSMLFIEEKN

**OXA-1072**  LELMSKEVKR VGYGNADIGT QVDNFWLVGP LKITPQQEAQ FAYKLANKTL PFSPKVQDEV QSMLFIEEKN

**OXA-1073**  LELMSKEVKR VGYGNADIGT QVDNFWLVGP LKITPQQEAQ FAYKLANNTL PFSPKVQDEV QSMLFIEEKN

**OXA-1074**  LELMSKEVKR VGYGNADIGT QVDNFWLVGP LKITPQQEAQ FAYKLANKTL PFSQKVQDEV QSMLFIEEKN

**OXA-1075**  LELMSNEVKR VGYGNADIGT QVDNFWLVGP LKITPQQEAQ FAYKLANKTL PFSQKVQDEV QSMLFIEEKN

**OXA-1076**  LELMSKEVKR VGYGNADIGT QVDNFWLVGP LKITPQQEAQ FAYKLANKTL PFSPKVQDEV QSMLFIEEKN

**OXA-1077**  LELMSNEVKR VGYGNADIGT QVDNFWLVGP LKITPQQEAQ FAYKLANKTL PFSQKVQDEV QSMLFIEEKN

**OXA-1078**  LELMSKEVKR VGYGNADIGT QVDNFWLVGP LKITPQQEAQ FAYKLANKTL PFSQKVQDEV QSMLFIEEKN

**OXA-1079**  LELMSKEVKR VGYSNADIGT QVDNFWLVGP LKITPQQEAQ FAYKLANKTL PFSQKVQDEV QSMLFIEEKN

**OXA-1080**  LELMSKEVKR VGYGNADIGT QVDNFWLVGP LKITPQQEAQ FAYKLANKTL PFSQKVQDEV QSMLFIEEKN

....|....| ....|....| ....|....| ....|....| ....|....| ....|....| ....|

220 230 240 250 260 270

**OXA-66**  GNKIYAKSGW GWDVNPQVGW LTGWVVQPQG NIVAFSLNLE MKKGIPSSVR KEITYKSLEQ LGIL*

**OXA-1058**  GNKIYAKSGW GWDVNPQVGW LTGWVVQPQG NIVAFSLNLE MKKGIPSSVR KEITYKSLEQ LGIL*

**OXA-1059**  GNKIYAKSGW GWDVNPQVGW LTGWVVQPQG NIVAFSLNLE MKKGIPSSVR KEITYKSLEQ LGIL*

**OXA-1060**  GNKIYAKSGW GWDVDPQVGW LTGWVVQPQG NIVAFSLNLE MKKGIPSSVR KEITYKSLEQ LGIL*

**OXA-1061**  GNKIYAKSGW GWDVNPQVGW LTGWVVQPQG NIVAFSLNLE MKKGIPSSVR KEITYKSLEK LGIL*

**OXA-1062**  GNKIYAKSGW GRDVNPQVGW LTGWVVQPQG NIVAFSLNLE MKKGIPSSVR KEITYKSLEQ LGIL*

**OXA-1063**  GNKIYAKSGW GRDVNPQVGW LTGWVVQPQG NIVAFSLNLE MKKGIPSSVR KEITYKSLEQ LGIL*

**OXA-1064**  GNKIYAQSGW GWDVNPQVGW LTGWVVQPQG NIVAFSLNLE MKKGIPSSVR KEITYKSLEQ LGIL*

**OXA-1065**  GNKIYAKSGW GWDVDPQVGW LTGWVVQPQG NIVAFSLNLE MKKGIPSSVR KEITYKSLEQ LGIL*

**OXA-1066**  GNKIYAKSGW GWDVNPQVGW LTGWVVQPQG NIVAFSLNLE MKKGIPSSVR KEITYKSLEQ LGIL*

**OXA-1067**  GNKIYAKSGW GWDVNPQVGW LTGWVVQPQG NIVAFSLNLE MKKGIPSSVR KEITYKSLEQ LGIL*

**OXA-1068**  GNKIYAKSGW GWDVNPQVGW LTGWVVQPQG NIVAFSLNLE MKKGIPSSVR KEITYKSLEQ LGIL*

**OXA-1069**  GNKIYAKSGW GWDVDPQVGW LTGWVVQPQG NIVAFSLNLE MKKGIPSSVR KEITYKSLEQ LGIL*

**OXA-1070**  GNKIYAKSGW GWDVNPQVGW LTGWVVQPQG NIVAFSLNLE MKKGIPSSVR KEITYKSLEQ LGIL*

**OXA-1071**  GNKIYAKSGW GWDVDPQVGW LTGWVVQPQG NIVAFSLNLE MKKGIPSSVR KEITYKSLEI LGIL*

**OXA-1072**  GNKIYAKSGW GWDVDPQVGW LTGWVVQPQG NIVAFSLNLE MKKGIPSSVR KEITYKSLEQ LDIL*

**OXA-1073**  GNKIYAKSGW GWDVDPQVGW LTGWVVQPQG NIVAFSLNLE MKKGIPSSVR KEITYKSLEQ LGIL*

**OXA-1074**  GNKIYAKSGW GWDVNPQVGW LTGWVVQPQG NIVAFSLNLE MKKGIPSSVR KEITYKSLEQ LGIL*

**OXA-1075**  GNKIYAESGW GWDVDPQVGW LTGWVVQPQG NIVAFSLNLE MKKGIPSSVR KEITYKSLEQ LGIL*

**OXA-1076**  GNKIYAKSGW GWDVDPQVGW LTGWVVQPQG NIVAFSLNLE MKKGIPSSVR KEITYKSLEQ LGIL*

**OXA-1077**  GNKIYAKSGW GWDVNPQVGW LTGWVVQPQG NIVAFSLNLE MKKGIPSSVR KEITYKSLEQ LGIL*

**OXA-1078**  GNKIYAKSGW GWDVDPQVGW LTGWVVQPQG NIVAFSLNLE MKKGIPSSVR KEITYKSLEQ LGIL*

**OXA-1079**  GNKIYAKSGW GWDVNPQVGW LTGWVVQPQG NIVAFSLNLE MKKGIPSSVR KEITYKSLEQ LGIL*

**OXA-1080**  GNKIYAKSGW GWDVNPQVGW LTGWVVQPQG NIVAFSLNLE MKKGIPSSVR KEITYKSLEQ LDIL*

**Supplementary Figure 2.** Multiple amino acid sequence alignment of the novel *bla*_OXA-51-like_ alleles with the *bla*_OXA-66_ leader sequence using ClustalW program in Bioedit v.7.2.5. Positions highlighted in yellow are those predicted to be conserved in the active site of the OXA-51 enzyme (Smith et al., 2015; doi:10.1021/acschembio.5b00090), while positions in blue are those predicted to promote carbapenem hydrolysis activity if mutated (Takebayashi et al., 2021; doi:10.1093/jac/dkaa502). Individual amino acids highlighted in green show the mutations of the enzymes relative to the leader sequence.
